# Proteolysis of adaptor protein Mmr1 during budding is necessary for mitochondrial homeostasis in *Saccharomyces cerevisiae*

**DOI:** 10.1038/s41467-022-29704-8

**Published:** 2022-04-14

**Authors:** Keisuke Obara, Taku Yoshikawa, Ryu Yamaguchi, Keiko Kuwata, Kunio Nakatsukasa, Kohei Nishimura, Takumi Kamura

**Affiliations:** 1 Laboratory of Biological Science, Division of Natural Science, Graduate School of Science, Nagoya University, Furo-cho, Chikusa-ku, Nagoya, 464-8602 Japan; 2 Institute of Transformative Bio-Molecules (ITbM), Nagoya University, Furo-cho, Chikusa-ku, Nagoya, 464-8601 Japan; 3 Graduate School of Science, Nagoya City University, Yamanohata 1, Mizuho-cho, Mizuho-ku, Nagoya, 467-8501 Japan

**Keywords:** Mitochondria, Ubiquitylation, Ubiquitin ligases

## Abstract

In yeast, mitochondria are passed on to daughter cells via the actin cable, motor protein Myo2, and adaptor protein Mmr1. They are released from the actin-myosin machinery after reaching the daughter cells. We report that Mmr1 is rapidly degraded by the ubiquitin-proteasome system in *Saccharomyces cerevisiae*. Redundant ubiquitin ligases Dma1 and Dma2 are responsible for Mmr1 ubiquitination. Dma1/2-mediated Mmr1 ubiquitination requires phosphorylation, most likely at S414 residue by Ste20 and Cla4. These kinases are mostly localized to the growing bud and nearly absent from mother cells, ensuring phosphorylation and ubiquitination of Mmr1 after the mitochondria enter the growing bud. In *dma1*Δ *dma2*Δ cells, transported mitochondria are first stacked at the bud-tip and then pulled back to the bud-neck. Stacked mitochondria in *dma1*Δ *dma2*Δ cells exhibit abnormal morphology, elevated respiratory activity, and increased level of reactive oxygen species, along with hypersensitivity to oxidative stresses. Collectively, spatiotemporally-regulated Mmr1 turnover guarantees mitochondrial homeostasis.

## Abstract (web summary)

Mitochondria are transported to daughter cells by myosin via an adaptor protein Mmr1 during budding in yeast. Here they show that the regulated proteolysis of Mmr1 after mitochondria inheritance is required for mitochondrial homeostasis.

## Introduction

Inheritance of organelles to the daughter cells and their proper distribution is essential for the homeostasis of eukaryotic cells. In the budding yeast *Saccharomyces cerevisiae*, organelles are transported into the newly emerging space, the bud, that eventually becomes an independent daughter cell. Mitochondria are transported into the growing bud by type-V myosin motor protein Myo2 on the actin cable^1,2^. Myo2 does not directly bind to mitochondria, but an adapter protein Mmr1 bridges the mitochondria and Myo2^2,3^. Mmr1 is proposed to bind to mitochondria through interaction with phospholipids in the outer mitochondrial membrane using basic amino acid residues^4^. In parallel to this system, mitochondria are passed on to the daughter cell via a pathway dependent on a small G-protein Ypt11^2,3^. Cells deleted for both *MMR1* and *YPT11* are inviable, although each of the single mutants is viable. Upon arrival at the bud, mitochondria are released from the actin-myosin machinery and are widely distributed in the daughter cells. Spatiotemporal regulation of the mitochondrial release from the actin-myosin machinery has not been defined so far. Proper distribution of mitochondria between mother and daughter cells via cell division is crucial for cell homeostasis because damaged mitochondria with low activity often generate more reactive oxygen species (ROS) that are toxic to cells. In *S. cerevisiae*, daughter cells preferentially inherit high-quality mitochondria that are more reducing and generate lower levels of ROS than those in mother cells^5^. Simultaneously, yeast cells are also equipped with a system in which a portion of high-quality mitochondria are anchored by Mfb1 and get reserved for the mother cell to avoid complete loss of high-quality mitochondria from mother cells^6,7^. Asymmetric inheritance of mitochondria during cell division is also vital for mammalian cells. For instance, the self-renewal of stem-like cells depends on the preferential inheritance of high-functioning young mitochondria, while older mitochondria tend to accumulate with somatic cell fates^8^. In humans, defects in mitochondrial localization and dynamics lead to diseases, highlighting the pivotal role of regulating mitochondrial dynamics^9–11^.

Proteomic analysis revealed that Mmr1 is a short-lived protein with a half-life of ~30 min^12^. The mechanism underlying the rapid turnover of Mmr1 and its relationship with mitochondrial inheritance remains unelucidated. In this study, we show that Mmr1 is degraded by the ubiquitin–proteasome system. The turnover of Mmr1 is dependent on Ste20- and Cla4-mediated phosphorylation and ubiquitination by redundant ubiquitin ligases Dma1 and Dma2. Ubiquitination and degradation of Mmr1 is required to release mitochondria from Myo2 after they entered the bud, maintain normal mitochondria morphology, and proper regulation of respiratory activity.

## Results

### Mmr1 is degraded by the proteasome

**Fig. 1:**
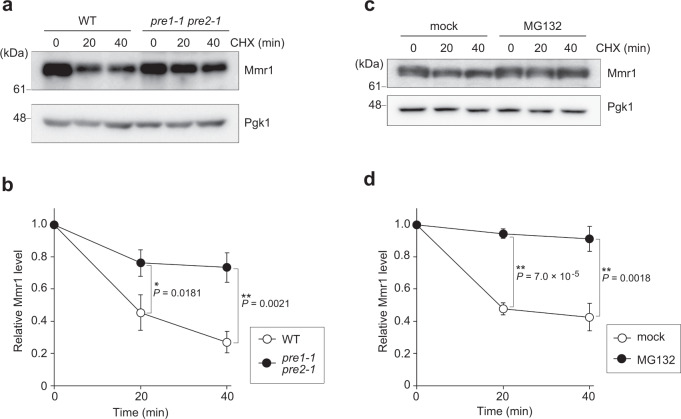
Mmr1 is degraded by the proteasome. **a** JB291 (WT) and JB290 (*pre1-1 pre2-1*) cells were grown to log phase in YPD medium at permissive temperature (25 °C), further incubated for 2 h at the restrictive temperature (37 °C), treated with cycloheximide (CHX), and harvested. Their total lysates were subjected to immunoblot analysis with anti-Mmr1 or, to demonstrate uniform loading, anti-Pgk1 antibody. **b** Mmr1 level was measured, normalized with that of Pgk1, and shown as a relative value to that at 0 min. **c** YTK4652 (*pdr5*Δ) cells were grown to log phase in YPD medium at 30 °C, treated with MG132 or mock-treated with dimethylsulfoxide for 40 min, treated with CHX, and harvested. Their total lysates were subjected to immunoblot analysis using anti-Mmr1 or anti-Pgk1 antibodies. **d** Mmr1 level was measured, normalized with that of Pgk1, and shown as a relative value to that at 0 min. Values in (**b**) and (**d**) represent the mean ± SD from three independent experiments. An unpaired two-tailed *t* test was used for the statistical analysis.

Proteomic analysis has revealed that Mmr1 is a short-lived protein^12^. We first investigated the mechanism behind the rapid turnover of Mmr1. Turnover of Mmr1 was monitored in the presence of cycloheximide (CHX) which inhibits de novo protein synthesis. In WT cells, Mmr1 was rapidly degraded upon CHX treatment, confirming that Mmr1 has a short life (Fig. 1a, b). In the *pre1-1 pre2-1* mutant, a temperature-sensitive mutant of proteasome subunits, turnover of Mmr1 was slower than that in WT cells at the restrictive temperature. Inhibition of the proteasome by a proteasome inhibitor MG132 also suppressed Mmr1 turnover (Fig. 1c, d). We confirmed that proteasome is indeed inhibited under these conditions (Supplementary Fig. 1). These results indicate that Mmr1 is rapidly degraded by the proteasome.

### Dma1 and Dma2 ubiquitinate Mmr1

**Fig. 2:**
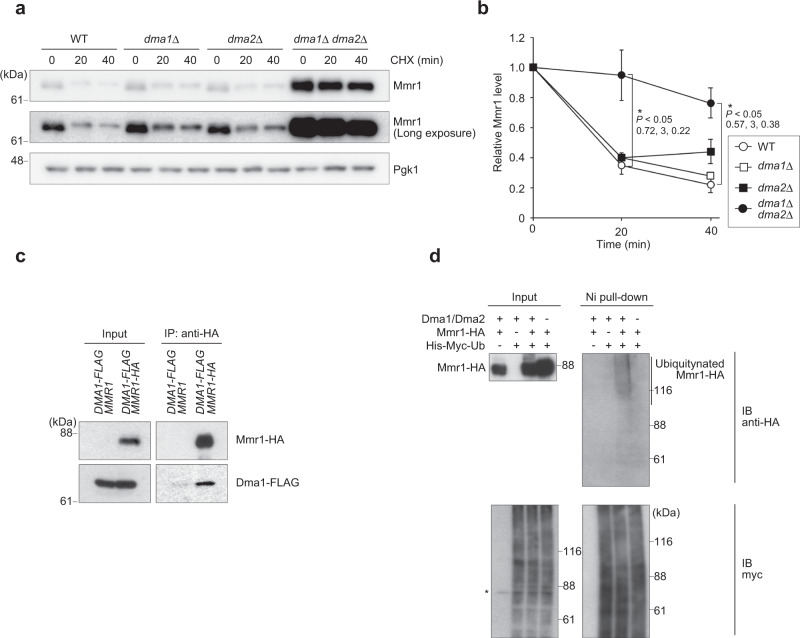
Dma1 and Dma2 are involved in Mmr1 turnover. **a** W303-1a (WT), YTK5414 (*dma1*Δ), YTK5415 (*dma2*Δ), and YTK5416 (*dma1*Δ *dma2*Δ) cells grown to log phase in YPD medium were treated with cycloheximide (CHX) and harvested. Their total lysates were prepared and subjected to immunoblot analysis with anti-Mmr1 or, to demonstrate uniform loading, anti-Pgk1 antibody. **b** Mmr1 level was measured, normalized with that of Pgk1, and shown as a relative value to that at 0 min. Values represent the mean ± SE from three independent experiments. Significance was tested by one-way analysis of variance (ANOVA) with Dunnett’s comparison (values of confidence intervals, degrees of freedom, and F are indicated). **c** YTK5423 (*cim3-1 DMA1-FLAG*) and YTK5425 (*cim3-1 DMA1-FLAG MMR1-HA*) cells were grown to log phase in YPD medium at permissive temperature (25 °C), further incubated for 2 h at the restrictive temperature (37 °C), and subjected to co-immunoprecipitation analysis using anti-HA antibody. The input and immunoprecipitated fraction were then subjected to immunoblot analysis with anti-HA or anti-FLAG antibodies. Similar results were obtained from two independent experiments. **d** YTK3487 (*cim3-1 MMR1-HA*), YTK6168 (*cim3-1 His-Myc-ubiquitin*), YTK5810 (*cim3-1 His-Myc-ubiquitin MMR1-HA*), and YTK5893 (*cim3-1 His-Myc-ubiquitin MMR1-HA dma1*Δ *dma2*Δ) cells were grown to log phase in YPD medium at permissive temperature (25 °C), further incubated for 90 min at the restrictive temperature (37 °C), and subjected to pull-down assay using Ni^2+^-agarose beads. The input and the pull-down fractions were then subjected to immunoblot analysis with anti-Myc or anti-HA antibodies. Similar results were obtained with two independent experiments. *, non-specific band.

Next, we explored an E3 ubiquitin ligase responsible for Mmr1 ubiquitination. To this end, HA-tagged Mmr1 was immunoprecipitated from *cim3-1* cells, a temperature-sensitive mutant of a proteasome subunit cultured at the restrictive temperature, and co-precipitated proteins were detected using liquid chromatography-tandem mass spectrometry (LC-MS/MS). Eight proteins annotated as E3 ubiquitin ligase, (Dma1, Dma2, Ubr1, Pep5, Grr1, Ubr2, Hul5, and Rsp5) were detected in the co-precipitated fraction (Supplementary Data 1 and Supplementary Table 1). Among them, double deletion of genes encoding redundant E3 ubiquitin ligases Dma1 and Dma2 resulted in prolonged life of Mmr1, while inactivation of other E3 ubiquitin ligases did not have this effect (Fig. 2a, b and Supplementary Fig. 2), indicating the critical role of Dma1 and Dma2 in Mmr1 turnover. We confirmed physical interaction between Dma1 and Mmr1 by co-immunoprecipitation followed by immunoblot analysis (Fig. 2c). In *cim3-1* background cells grown at the restricted temperature, ubiquitinated Mmr1 was accumulated in a manner dependent on Dma1 and Dma2 (Fig. 2d). Collectively, it is evident that Dma1 and Dma2 are crucially involved in Mmr1 ubiquitination.

### Dma1 and Dma2 are required for normal mitochondrial dynamics

**Fig. 3:**
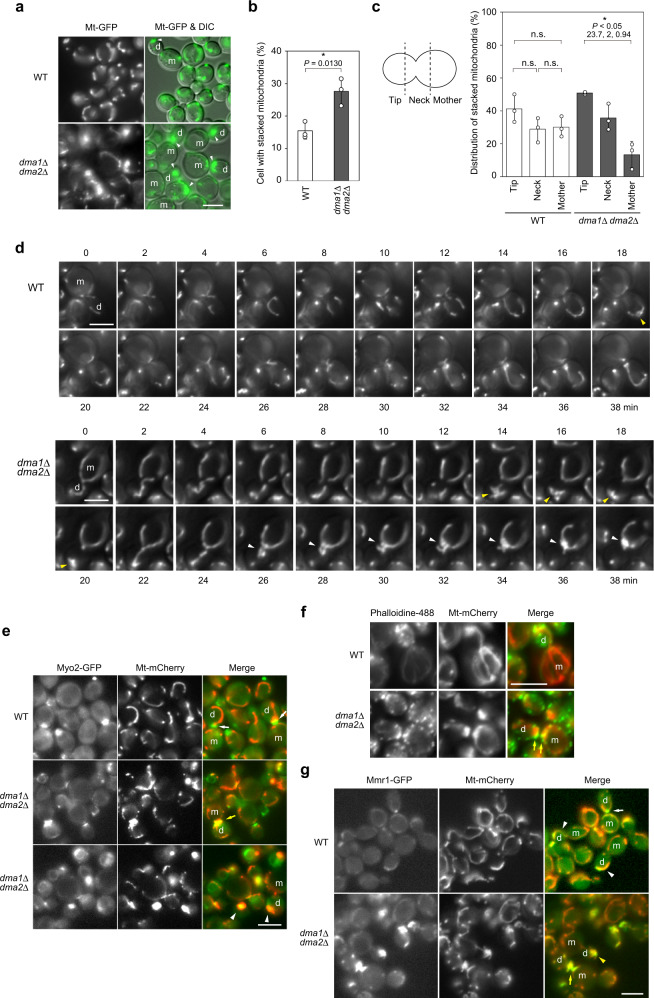
Dma1 and Dma2 are required for the correct positioning of mitochondria. **a** YTK5334 (*Mt-GFP*) and YTK5341 (*Mt-GFP dma1*Δ *dma2*Δ) cells grown to log phase in a synthetic complete medium were observed by fluorescence microscopy. Arrowhead, stacked mitochondria. **b** Proportion of cells with stacked mitochondria. **c** Distribution of stacked mitochondria. **d** YTK5334 (*Mt-GFP*) and YTK5341 (*Mt-GFP dma1*Δ *dma2*Δ) cells grown to log phase in synthetic complete medium were serially observed by fluorescence microscopy using glass-base dishes. Yellow and white arrowheads, stacked mitochondria at bud tip and the bud neck, respectively. **e** YOK5303 (*Mt-mCherry MYO2-GFP*) and YOK5304 (*Mt-mCherry MYO2-GFP dma1*Δ *dma2*Δ) cells grown to log phase in synthetic complete medium were subjected to fluorescence microscopy. White and yellow arrow, co-localization of Myo2-GFP with a part of mitochondria at the bud neck in WT cells and entire mitochondria at the bud neck in *dma1*Δ *dma2*Δ cells, respectively. Arrowhead, Myo2-GFP co-localized with mitochondria and mislocalized to an abnormal site in *dma1*Δ *dma2*Δ cells. **f** YOK5182 (*Mt-mCherry*) and YOK5185 (*Mt-mCherry dma1*Δ *dma2*Δ) cells grown to log phase in YPD medium were fixed and subjected to F-actin visualization. Yellow arrow, F-actin co-localizing or adjacent to stacked mitochondria at bud neck. **g** YOK5595 (*Mt-mCherry MMR1-GFP*) and YOK5596 (*Mt-mCherry MMR1-GFP dma1*Δ *dma2*Δ) cells grown to log phase in a synthetic complete medium were observed by fluorescence microscopy. White arrow, partial co-localization of Mmr1 and a part of mitochondria at the bud neck in WT cells. White arrowhead, co-localization of Mmr1 and tubular mitochondria during transportation to daughter cell. Yellow arrow, accumulation of Mmr1 on stacked mitochondria at bud neck in *dma1*Δ *dma2*Δ cells. Yellow arrowhead, accumulation of Mmr1 on stacked mitochondria at bud tip in *dma1*Δ *dma2*Δ cells. Bar, 5 µm (for all panels). m and d mother and daughter cells, respectively (for all panels). Values in (**b**) and (**c**) represent means ± SDs from three independent experiments. An unpaired two-tailed *t* test was used for statistical analysis in (**b**). In (**c**), significance was tested by one-way analysis of variance with Tukey’s comparison (values of confidence intervals, degrees of freedom, and F are indicated) for each strain. n.s., not significant. For (**d**–**g**), similar results were obtained from three independent experiments.

In order to investigate the relationship between Dma1/2-mediated turnover of Mmr1 and mitochondria inheritance, mitochondrial dynamics were monitored using fluorescent proteins fused to the mitochondrial presequence of F_0_-ATPase subunit 9 (Su9 encoded by *OLI1*)^13^. In WT cells, tubular mitochondria were distributed throughout the mother and daughter cells (Fig. 3a). In contrast, in a significant portion of *dma1*Δ *dma2*Δ cells, mitochondria were stacked at the bud tip or at the bud neck, suggesting that Dma1 and Dma2 are required for the correct distribution of mitochondria (Fig. 3a, b). Mitochondria sometimes appeared to be stacked even in WT cells, but the apparent mitochondrial stacking in WT cells was just a transient phenomenon during the continuous dynamic movement of mitochondria, as observed using time-lapse imaging (Supplementary Movie 1 and Supplementary Fig. 3). Likewise, stacked mitochondria were evenly observed throughout WT daughter and mother cells in snapshot micrographs, while stacked mitochondria were enriched at the bud tip and the bud neck in *dma1*Δ *dma2*Δ cells (Fig. 3c). To investigate the kinetics of mitochondria transport, we performed a serial observation of mitochondria. In WT cells, mitochondria were transported into the growing bud, and, subsequently, the tubular mitochondria widely covered the daughter cell (Fig. 3d, Supplementary Movie 1, and Supplementary Fig. 3). In contrast, in *dma1*Δ *dma2*Δ cells, mitochondria were transported into the growing bud and stacked at the bud tip, but they soon moved back to the bud neck where they were stacked again (Fig. 3d, Supplementary Movie 2, and Supplementary Fig. 3). Therefore, the difference in the position of stacked mitochondria in *dma1*Δ *dma2*Δ cells, *i.e*., bud tip and bud neck, observed in snapshot micrographs represents the difference in the stage of mitochondria transport. A striking feature of mitochondria transport in *dma1*Δ *dma2*Δ cells was their backward movement from the bud tip to the bud neck and reminiscent of that of Myo2. As Myo2 moves along the actin cable toward the bud tip, it first accumulates at the bud tip during bud growth. However, at the cytokinesis stage, Myo2 transports vesicles toward the bud neck and, therefore, accumulates at the bud neck^14^. To investigate the relationship between the Myo2 translocation to the bud neck and mitochondrial stacking in *dma1*Δ *dma2*Δ cells, we performed double-labeling of Myo2 and mitochondria. In WT cells, only a part of the total mitochondria (often at one end of the tubular mitochondria) co-localized with Myo2 at the bud neck (white arrow in Fig. 3e), resulting in the release of the majority of the mitochondria from Myo2. In *dma1*Δ *dma2*Δ cells, all the mitochondria stacked at the bud neck (67 out of 67) were co-localized with Myo2. As against the partial co-localization of mitochondria and Myo2 at the bud neck in WT cells, almost the entire part of the stacked mitochondria was co-localized with Myo2 at the bud neck in *dma1*Δ *dma2*Δ cells (yellow arrow in Fig. 3e). These observations strongly suggest that Mmr1 degradation following the Dma1- and Dma2-mediated ubiquitination is required to release the majority of mitochondria from Myo2 and thereby guarantees their dynamic movement in the daughter cell. In contrast, we sometimes observed mislocalized and stacked Myo2 in the cytosol in *dma1*Δ *dma2*Δ cells (arrowhead in Fig. 3e). The stacked Myo2 co-localized well with the stacked mitochondria, suggesting that the localization of Myo2 is sometimes disturbed by stacked mitochondria that failed to be released from Myo2. Therefore, it is likely that the release of mitochondria from Myo2 through Dma1/2-mediated degradation of Mmr1 guarantees the correct positioning of both mitochondria and Myo2. F-actin was always localized adjacent to the stacked mitochondria at the bud neck in *dma1*Δ *dma2*Δ cells, often at the bud-neck side of mitochondria (yellow arrow in Fig. 3f) (63 stacked mitochondria at the bud neck out of 63 were accompanied by the F-actin signal). F-actin located adjacent to the stacked mitochondria in *dma1*Δ *dma2*Δ cells appeared as an actin-patch rather than actin-cables. Since mitochondria are transported into the daughter cells on these actin-cables, Myo2 molecules that carried mitochondria to the bud tip would be getting translocated to the bud neck along with the mitochondria after getting dissociated from the actin cable and gets attached to the actin-patch there. We also investigated the localization of Mmr1. We found that endogenously-expressed Mmr1-GFP localized to mitochondria during transportation to the bud in WT (white arrowhead in Fig. 3g). When mitochondria reached the bud tip, Mmr1 mildly accumulated at the tip. Mmr1 also localized to some extent at the bud neck during the cytokinesis stage, and a part of mitochondria was co-localized similar to Myo2-GFP (white arrow in Fig. 3g; see Discussion). In *dma1*Δ *dma2*Δ cells, Mmr1 heavily accumulated at the bud tip and bud neck where mitochondria were stacked (yellow arrowhead and yellow arrow, respectively, in Fig. 3g). Stacked mitochondria at the bud neck of *dma1*Δ *dma2*Δ cells were always co-localized well with Mmr1 (50 out of 50). These observations strongly suggest that Dma1- and Dma2-dependent ubiquitination and following degradation of Mmr1 mediate the dissociation of mitochondria from Myo2 to prevent their stacking at the bud tip and the bud neck.

### Dma1/2 are required for normal mitochondrial morphology

**Fig. 4:**
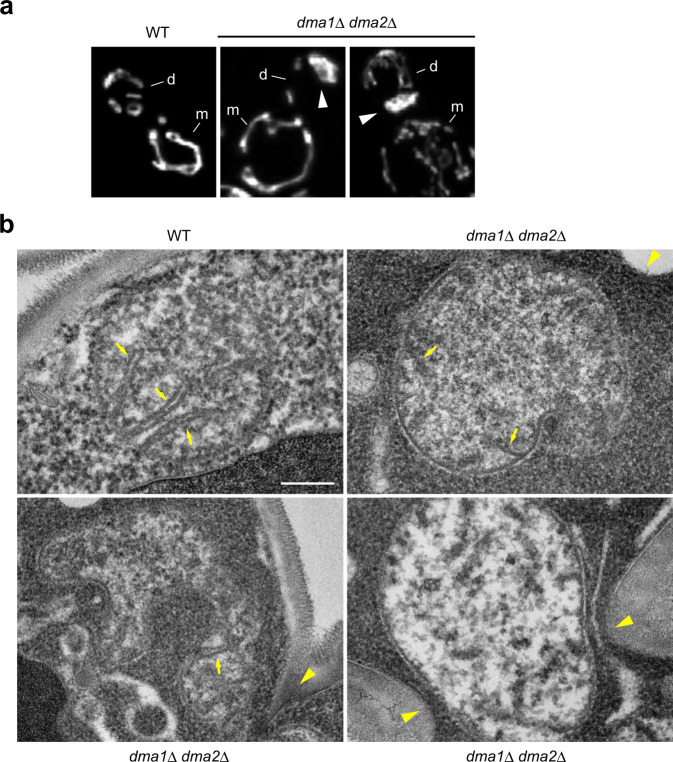
Dma1 and Dma2 are required for the maintenance of mitochondria morphology. **a** YTK5334 (*Mt-GFP*) and YTK5341 (*Mt-GFP dma1*Δ *dma2*Δ) cells were grown to log phase in synthetic complete medium, fixed, and observed under a fluorescence microscope. Optical sections were obtained using a structured-illumination microscopy system at an interval of 0.2 µm, deconvoluted, and reconstructed into a 3D image. m and d, mother cell and daughter cell, respectively. Arrowhead, stacked mitochondria. **b** W303-1a (WT) and YTK5416 (*dma1*Δ *dma2*Δ) cells were grown to log phase in a synthetic complete medium and subjected to electron microscopy. 58 and 54 cells for WT and *dma1*Δ *dma2*Δ cells, respectively, were analyzed. Arrow, cristae. Arrowhead, bud neck. Bar, 200 nm.

Next, we analyzed the structure of stacked mitochondria in *dma1*Δ *dma2*Δ cells in detail using 3D reconstruction of fluorescence images and electron microscopy. First, optical sections of Mt-GFP fluorescence images were obtained via structured-illumination microscopy and reconstructed into 3D images. This observation highlighted intricately entwined mitochondria at the bud tip and bud neck in *dma1*Δ *dma2*Δ cells (Fig. 4a and Supplementary Movies 3–5). In electron microscopy, WT mitochondria exhibited a clear round shape containing cristae in the cross-section (Fig. 4b). In contrast, mitochondria at the bud neck of *dma1*Δ *dma2*Δ cells were often expanded or deformed into abnormal morphology, although the cristae structures could still be observed. These observations indicate that Dma1/2-mediated degradation of Mmr1 is required for normal mitochondria morphology in addition to their correct positioning.

### Phosphorylation regulates the timing of Mmr1 degradation

**Fig. 5:**
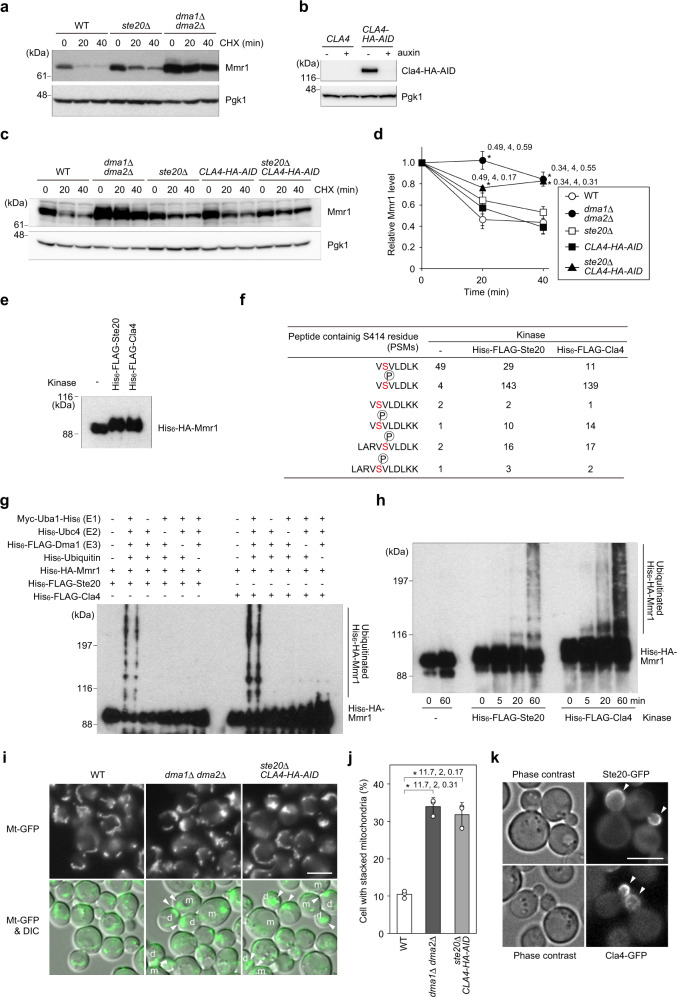
Ste20 and Cla4 are involved in Mmr1 turnover. **a** W303-1a (WT), YTK5879 (*ste20*Δ), and YTK5416 (*dma1*Δ *dma2*Δ) cells in log phase were treated with cycloheximide (CHX) and harvested. Total lysates were subjected to immunoblotting. **b** BY25598 (*CLA4*) and YOK5411 (*CLA4-HA-AID*) cells in log phase were treated with or without 3-indoleacetic acid (auxin) for 3 h and harvested. Total lysates were subjected to immunoblotting. **c** BY25598 (WT), YTK5416 (*dma1*Δ *dma2*Δ), YTK5879 (*ste20*Δ), YOK5411 (*CLA4-HA-AID*), and YOK5409 (*ste20*Δ *CLA4-HA-AID*) cells in log phase were treated with 3-indoleacetic acid for 3 h, treated with CHX, and harvested. Total lysates were subjected to immunoblotting. **d** Mmr1 levels were measured, normalized to those of Pgk1, and shown as a relative value to that at 0 min. **e** Purified His_6_-HA-Mmr1 was incubated with or without either purified His_6_-FLAG-Ste20 or His_6_-FLAG-Cla4 for 1 h at 30 °C, and subjected to immunoblotting. **f** Number of detected peptides (PSMs) containing S414 residue of Mmr1, phosphorylated or not, in MS analysis after in vitro phosphorylation. The Ser residue corresponding to S414 residue of Mmr1 is red. Circled P indicates phosphorylation. **g** Purified His_6_-HA-Mmr1 was incubated with either purified His_6_-FLAG-Ste20 or His_6_-FLAG-Cla4 for 1 h at 30 °C. Phosphorylated His_6_-HA-Mmr1 was then subjected to ubiquitination assays and analyzed by immunoblotting. **h** Purified His_6_-HA-Mmr1 was mock-treated or treated with either purified His_6_-FLAG-Ste20 or His_6_-FLAG-Cla4 for 1 h at 30 °C. After the in vitro phosphorylation, His_6_-HA-Mmr1 was subjected to ubiquitination reaction and analyzed by immunoblotting. **i** YTK5334 (*Mt-GFP*), YTK5341 (*Mt-GFP dma1*Δ *dma2*Δ), and YOK5434 (*Mt-GFP ste20*Δ *CLA4-HA-AID*) cells were grown to log phase with 3 h treatment with 3-indoleacetic acid and observed. m and d, mother and daughter cells, respectively. Arrowhead, stacked mitochondria. Bar, 5 µm. **j** Proportion of cells with stacked mitochondria. **k** YOK5305 (*STE20-GFP*) and YOK5407 (*CLA4-GFP*) cells in log phase were observed. Arrowhead, GFP signal at the cortex of daughter cell. Bar, 5 µm. **d**, **j** Values represent the means ± SDs from three independent experiments. Significance was tested by one-way analysis of variance with Dunnett’s (**d**) or Tukey’s (**j**) comparison (values of confidence intervals, degrees of freedom, and F are indicated). *P < 0.05. Similar results were obtained with three independent experiments.

To ensure the inheritance of mitochondria to daughter cells, mitochondria should be released from Myo2 only after they have entered the growing bud. Therefore, Dma1/2-mediated degradation of Mmr1 should be strictly regulated spatially and temporally. Since Dma1/2 are considered to recognize phosphorylated substrates using their FHA domains^15,16^, we first searched for the protein kinase involved in Mmr1 turnover. Our protein MS analysis of the Mmr1-HA pull-down fraction revealed that the S414 residue of Mmr1 is phosphorylated (Supplementary Data 1 and Supplementary Fig. 4). The S414 residue matched with the consensus phosphorylation motifs, namely the RxS sequence of the p21-activated kinase family, to which Ste20 and Cla4 belong^17–19^. Therefore, we first focused on these kinases and examined their involvement in Mmr1 turnover. To this end, we monitored Mmr1 turnover in *ste20*Δ cells. Mmr1 turnover appeared to be slightly delayed in *ste20*Δ cells, suggesting the potential involvement of Ste20 in Mmr1 turnover (Fig. 5a). However, Mmr1 degradation was still faster in *ste20*Δ cells than in *dma1*Δ *dma2*Δ cells, and therefore, we went on to examine Mmr1 turnover in double-mutant cells lacking kinase activity of both Ste20 and Cla4. Since *ste20*Δ *cla4*Δ double-mutant cells are inviable^20^, we utilized the auxin-inducible degron (AID) system in which AID-tagged proteins are transiently degraded upon treatment with a phytohormone auxin^21^. We confirmed that Cla4-HA-AID was successfully degraded by auxin treatment (Fig. 5b). In auxin-treated *ste20*Δ *CLA4-HA-AID* cells, Mmr1 degradation was slower than those in auxin-treated WT, *ste20*Δ, and *CLA4-HA-AID* cells (Fig. 5c, d), indicating the involvement of both Ste20 and Cla4 in Mmr1 turnover. The level of Mmr1 at time 0 was higher in *dma1*Δ *dma2*Δ cells than in *ste20*Δ *CLA4-HA-AID* cells for unknown reasons. To examine the direct involvement of Ste20 and Cla4 in Mmr1 turnover, we established an in vitro phosphorylation and ubiquitination system of Mmr1. First, in vitro phosphorylation of purified 6-histidine (His_6_)-HA-Mmr1 by purified His_6_-FLAG-Ste20 or His_6_-FLAG-Cla4 was examined. Incubation of His_6_-HA-Mmr1 with either of the recombinant kinases caused a reduction in His_6_-HA-Mmr1 mobility in SDS-PAGE (Fig. 5e). MS analysis revealed that the number of detected peptides containing phosphorylated S414 residue of Mmr1 was increased following incubation with His_6_-FLAG-Ste20 or His_6_-FLAG-Cla4, while that of peptides containing unphosphorylated S414 residue of Mmr1 did not increase (Fig. 5f). These results indicate that Ste20 and Cla4 phosphorylate the S414 residue of Mmr1, at least in vitro. Next, we examined in vitro ubiquitination of phosphorylated His_6_-HA-Mmr1 by adding purified E1 (Myc-Uba1-His_6_), E2 (His_6_-Ubc4), E3 (His_6_-FLAG-Dma1), and ubiquitin (His_6_-ubiquitin). In this reconstitution system, purified His_6_-HA-Mmr1 was ubiquitinated depending on E1, E2, E3, ubiquitin, and pretreatment with either His_6_-FLAG-Ste20 or His_6_-FLAG-Cla4 (Fig. 5g, h), indicating the critical role of Ste20 and Cla4 in Dma1/2-mediated Mmr1 ubiquitination. Next, we analyzed the dynamics of mitochondria in auxin-treated *ste20*Δ *CLA4-HA-AID* cells. Mitochondria stacked at the bud tip and bud neck in auxin-treated *ste20*Δ *CLA4-HA-AID* cells, similar to that in *dma1*Δ *dma2*Δ cells, implying that Ste20 and Cla4 are required for normal mitochondria distribution (Fig. 5i, j).

To ensure proper mitochondrial inheritance to daughter cells, Mmr1 should be degraded only after the mitochondria have entered the growing bud. We hypothesized that Ste20- and Cla4-mediated phosphorylation of Mmr1 is a key regulatory step determining the timing of Mmr1 degradation. To verify this, we observed the localization of chromosomally expressed Ste20-GFP and Cla4-GFP. Both Ste20-GFP and Cla4-GFP were mostly detected at the bud cortex and were nearly undetectable in mother cells (Fig. 5k) (we observed more than 300 cells for each protein and confirmed their negligible localization to the mother cell). This restricted localization of Ste20 and Cla4 is consistent with previous reports^22,23^, and maybe a mechanism to ensure that Mmr1 is degraded only after the mitochondria have reached the growing bud.

### S414 residue of Mmr1 regulates its turnover

**Fig. 6:**
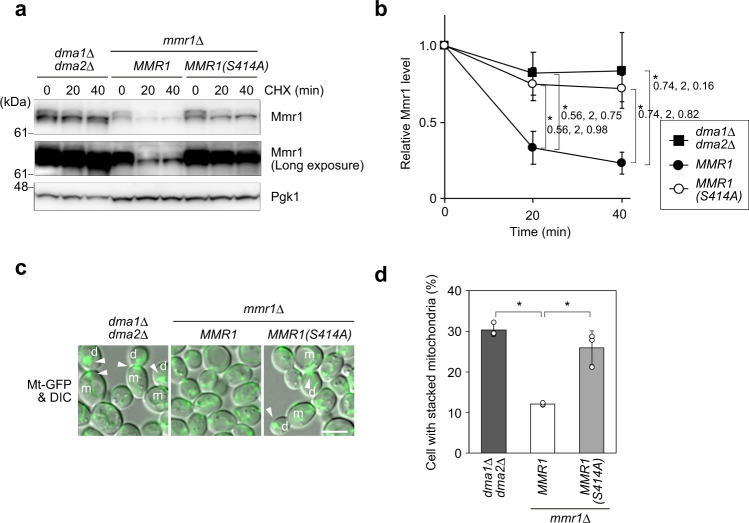
Ser414 residue in Mmr1 is important for its rapid turnover. **a** YTK5416 (*dma1*Δ *dma2*Δ), YTK5675 (*mmr1*Δ *MMR1*), and YTK5764 [*mmr1*Δ *MMR1(S414A)*] cells were grown to log phase in YPD medium, treated with CHX, and harvested. Their total lysates were subjected to immunoblot analysis with anti-Mmr1 or, to demonstrate uniform loading, anti-Pgk1 antibody. **b** Mmr1 level was measured, normalized with that of Pgk1, and shown as a relative value to that at 0 min. **c** YTK5341 (*Mt-GFP dma1*Δ *dma2*Δ), YOK5455 (*Mt-GFP mmr1*Δ *MMR1*), and YOK5456 [*Mt-GFP mmr1*Δ *MMR1(S414A)*] cells were grown to log phase in synthetic complete medium and observed under a fluorescence microscope. m and d, mother cell and daughter cell, respectively. Arrowhead, stacked mitochondria. Bar, 5 µm. **d** Proportion of cells with stacked mitochondria was calculated. Values in (**b**) and (**d)** represent the mean ± SD from three independent experiments. Significance was tested by one-way analysis of variance with Dunnett’s comparison (values of confidence intervals, degrees of freedom, and F are indicated) (**b)** or Steel’s comparison test (**d**). *P < 0.05.

Given the important role of Ste20 and Cla4 in Mmr1 turnover, we searched for the critical phosphorylation site in Mmr1. In addition to the motif including the S414 residue, Mmr1 contains three other RxS consensus phosphorylation motifs for Ste20 and Cla4 (Supplementary Fig. 4b). We substituted each Ser residue in the RxS motifs with Ala and monitored the turnover of the resultant mutant Mmr1 proteins, expressed from their own promoter, via CHX chase experiments. Among the four mutant Mmr1 proteins, only Mmr1-S414A had a prolonged life compared to unmutated Mmr1 (Fig. 6a, b and Supplementary Fig. 5). In *mmr1*Δ cells expressing the Mmr1-S414A mutant protein, mitochondria were stacked at the bud tip and bud neck, similar to that in *dma1*Δ *dma2*Δ cells (Fig. 6c, d). These results indicate that phosphorylation of Mmr1, most likely at S414 residue, is critical for the rapid turnover of Mmr1 and, thereby, for the normal distribution of the mitochondria.

### Mmr1 degradation enables increased tolerance to ROS

**Fig. 7:**
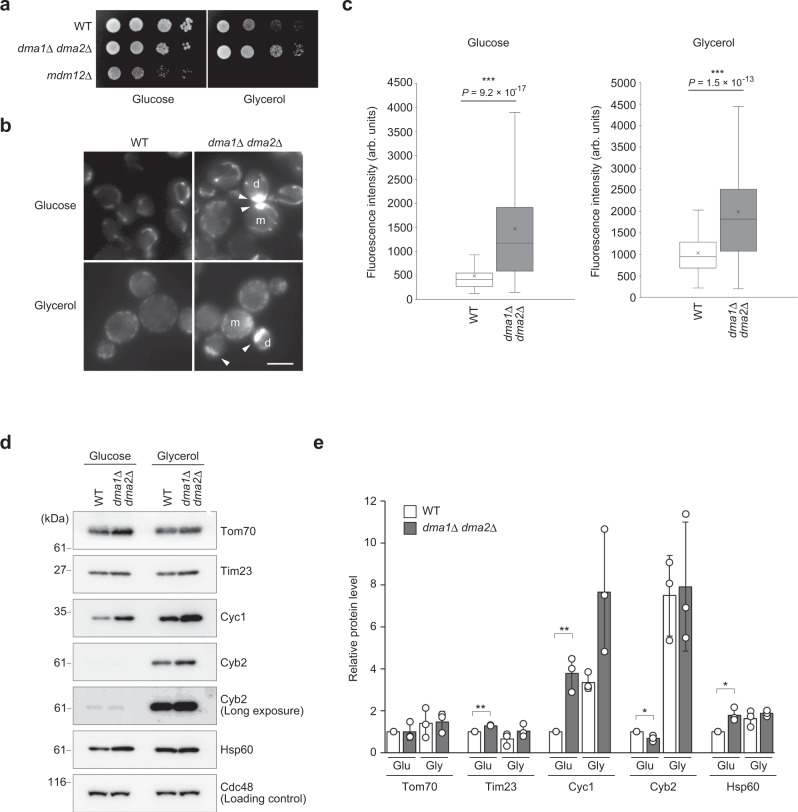
Mitochondria in *dma1*Δ *dma2*Δ cells have elevated respiratory activity. **a** W303-1a (WT), YTK5416 (*dma1*Δ *dma2*Δ), and YTK5433 (*mdm12*Δ) cells were grown to log phase; the cell density of each suspension culture was adjusted to *A*_600_ = 0.5 and serially diluted to 1:10, and spotted on YPD (Glucose) or YPGlycerol (Glycerol) plate in which glycerol was used as the carbon source instead of glucose, and incubated at 30 °C for 48 h. **b** W303-1a (WT) and YTK5416 (*dma1*Δ *dma2*Δ) cells were grown to log phase in a synthetic complete medium (either glucose or glycerol was used as the carbon source), loaded with 50 nM TMRM for 1 h, and observed by fluorescence microscopy. Arrowhead, TMRM signal from stacked mitochondria. m and d, mother and daughter cells, respectively. **c** Intensity of TMRM signal. Imaging data from (**b**) were used for the analysis. The box covers the region from the 1st quartile to the 3rd quartile. The horizontal line and cross mark represent the median and the mean, respectively. The whiskers at either side of the box extend to 1.5 interquartile ranges from the quartiles. For cells grown in glucose-containing medium, 103 and 112 cells for WT and *dma1*Δ *dma2*Δ, respectively, were analyzed. For cells grown in glycerol-containing medium, 117 and 113 cells for WT and *dma1*Δ *dma2*Δ, respectively, were analyzed. Significance was tested by Mann–Whitney’s *U* test. Similar results were obtained from two independent experiments. **d** W303-1a (WT) and YTK5416 (*dma1*Δ *dma2*Δ) cells were grown to log phase in YPD (Glucose) or YPGlycerol (Glycerol) medium, in which glycerol was used as the carbon source instead of glucose, and harvested. Total lysates were subjected to immunoblotting with anti-Tom70, anti-Tim23, anti-Cyc1, anti-Cyb2, anti-Hsp60 antibodies, or to demonstrate uniform loading, an anti-Cdc48 antibody. **e** Protein levels in (**d**) were measured, normalized to those of Cdc48, and shown as a relative value to that in WT cells grown in YPD medium. Values represent the mean ± SD from three independent experiments. An unpaired two-tailed *t* test followed by Bonferroni correction was used for statistical analysis. * and **P < 0.05 and *P* < 0.01, respectively.

Next, we investigated the physiological role of regulated Mmr1 turnover. Given the abnormal morphology of mitochondria in *dma1*Δ *dma2*Δ cells (Fig. 4), we hypothesized that respiratory activity would be altered in *dma1*Δ *dma2*Δ cells. To examine this hypothesis, the growth of *dma1*Δ *dma2*Δ cells was compared with that of WT cells on plates containing glucose or glycerol as a carbon source. When fermentable carbon sources such as glucose are available, yeast cells mainly generate ATP by glycolysis that occurs in the cytosol. In contrast, ATP synthesis depends on oxidative phosphorylation occurring in the mitochondria when carbon sources are non-fermentable ones like glycerol. Therefore, mutants with reduced respiratory activity exhibit a slow-growth phenotype or lethality on plates containing non-fermentable carbon sources. On plates containing glucose, *dma1*Δ *dma2*Δ cells showed slight growth retardation compared to WT cells (Fig. 7a). However, *dma1*Δ *dma2*Δ cells grew faster than WT cells on glycerol plates, suggesting that respiratory activity was elevated in this mutant. To evaluate respiratory activity, mitochondria were labeled with TMRM fluorescent reagent that gets incorporated into the mitochondria depending on mitochondrial membrane potential, which roughly reflects respiratory activity^24^. Mitochondria of *dma1*Δ *dma2*Δ cells emitted more intense fluorescence than those of WT cells both in glucose- and glycerol-containing media, indicating that *dma1*Δ *dma2*Δ mitochondria possessed elevated membrane potential (Fig. 7b, c). This notion was further supported by immunoblot analysis showing an elevated level of cytochrome c (Cyc1), an electron carrier in the electron-transfer chain system, in *dma1*Δ *dma2*Δ cells in both glucose- and glycerol-containing media (Fig. 7d, e). These results strongly suggest that *dma1*Δ *dma2*Δ cells had higher respiratory activity than WT cells, which in turn suggests that Dma1/2-mediated Mmr1 turnover influences the regulation of respiratory activity.

**Fig. 8:**
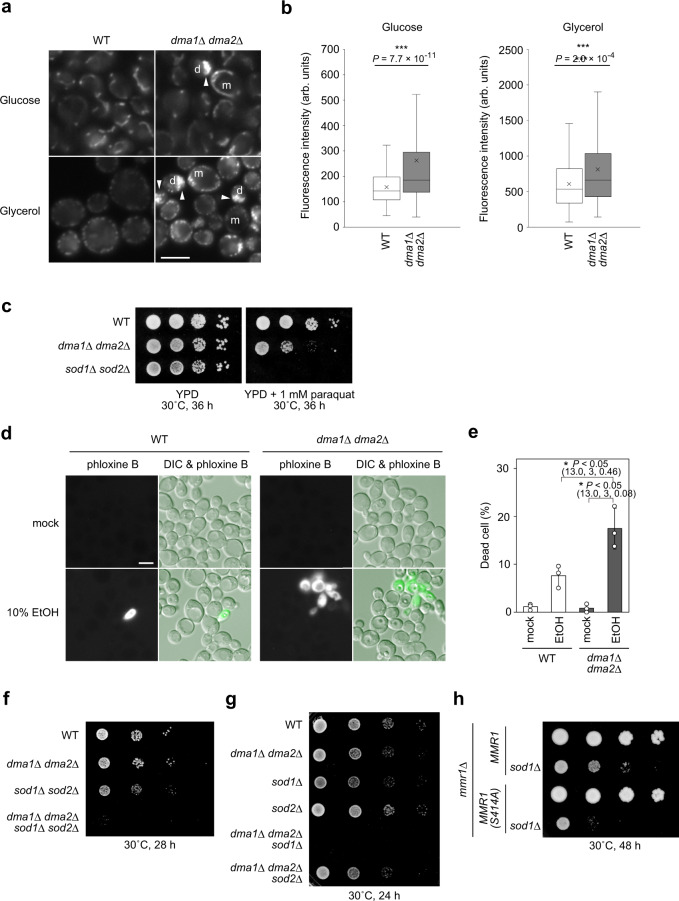
*dma1*Δ *dma2*Δcells produce more ROS. **a** W303-1a (WT) and YTK5416 (*dma1*Δ *dma2*Δ) cells in log phase were loaded with 5 µM CellROX Green reagent for 1 h, and observed by fluorescence microscopy. m and d, mother and daughter cells, respectively. Arrowhead, CellROX Green reagent signal from stacked mitochondria. Bar, 5 µm. **b** Intensity of CellROX Green reagent signal. Imaging data from (**a**) were used. The box covers the region from the 1st quartile to the 3rd quartile. The horizontal line and cross mark represent the median and the mean, respectively. The whiskers at either side of the box extend to 1.5 interquartile ranges from the quartiles. For glucose-containing medium, 224 and 267 cells for WT and *dma1*Δ *dma2*Δ, respectively, were analyzed. For glycerol-containing medium, 180 and 261 cells for WT and *dma1*Δ *dma2*Δ, respectively, were analyzed. Significance was tested by Mann–Whitney’s *U* test. Similar results were obtained from two independent experiments. **c** W303-1a (WT), YTK5416 (*dma1*Δ *dma2*Δ), and YOK5468 (*sod1*Δ *sod2*Δ) cells were grown to log phase; the cell density of each suspension culture was adjusted to *A*_600_ = 0.5, serially diluted to 1:10, and spotted on YPD with or without 1 mM paraquat. **d** W303-1a (WT) and YTK5416 (*dma1*Δ *dma2*Δ) cells in log phase were treated with 10% ethanol (EtOH) or mock-treated for 2 h, stained with 8 µg/mL phloxine B for 5 min, and observed. Bar, 5 µm. **e** Proportions of dead cells were calculated using imaging data from (**d**). Values represent the means ± SDs from three independent experiments. Significance was tested by one-way analysis of variance with Tukey’s comparison (values of confidence intervals, degrees of freedom, and F are indicated). **f** W303-1a (WT), YTK5416 (*dma1*Δ *dma2*Δ), YOK5468 (*sod1*Δ *sod2*Δ), and YOK5481 (*dma1*Δ *dma2*Δ *sod1*Δ *sod2*Δ) cells were treated as in (**c**) and spotted on YPD medium. **g** W303-1a (WT), YTK5416 (*dma1*Δ *dma2*Δ), YOK5464 (*sod1*Δ), YOK5466 (*sod2*Δ), YOK5479 (*dma1*Δ *dma2*Δ *sod1*Δ), and YOK5480 (*dma1*Δ *dma2*Δ *sod2*Δ) cells were treated as in (**c**) and spotted on YPD medium. **h** YOK5482 (*mmr1*Δ *MMR1*), YOK5485 (*mmr1*Δ *MMR1 sod1*Δ), YOK5483 [*mmr1*Δ *MMR1(S414A)*], and YOK5486 [*mmr1*Δ *MMR1(S414A) sod1*Δ] cells were treated as in (**c**) and spotted on YPD medium.

Dysregulation of respiratory activity is often accompanied by cytotoxicity caused by increased generation of ROS^25,26^. To monitor ROS levels, cells were labeled with CellROX Green FM reagent. This cell-permeable reagent is nonfluorescent in a reduced state but emits a fluorogenic signal upon oxidation by ROS. Oxidized CellROX Green reagent binds to DNA, thereby primarily labeling mitochondria generating ROS, and is successfully detected using fluorescence microscopy^27^. Growing WT and *dma1*Δ *dma2*Δ cells were loaded with CellROX Green reagent and subjected to fluorescence microscopy. As shown in Fig. 8a, b, *dma1*Δ *dma2*Δ cells emitted more intense fluorescence signals from their mitochondria, particularly the stacked ones, than WT cells, both in glucose- and glycerol-containing media. This observation clearly reveals that *dma1*Δ *dma2*Δ cells generate more ROS than WT cells. Cells expressing the Mmr1-S414A variant also showed higher emission of fluorescence after adding TMRM and CellRox Green FM reagents compared to cells expressing unmutated Mmr1 (Supplementary Fig. 6), indicating that the elevation in respiratory activity and ROS level in *dma1*Δ *dma2*Δ cells is caused by defective turnover of Mmr1 but not due to the pleiotropic effect of *DMA1* and *DMA2* deletion.

Next, we attempted to determine if elevated ROS production rendered *dma1*Δ *dma2*Δ cells hypersensitive to oxidative stress. Consequently, we examined the growth of WT and *dma1*Δ *dma2*Δ cells on plates containing paraquat, an ROS generating reagent. *dma1*Δ *dma2*Δ cells exhibited hypersensitivity to paraquat (Fig. 8c). We also examined the sensitivity of *dma1*Δ *dma2*Δ cells to ethanol, which is also known to induce ROS production^28^. Cell viability was evaluated by staining cells with phloxine B, which is actively pumped out from living cells but is deposited on dead cells and emits intense fluorescence. Ethanol treatment caused the death of *dma1*Δ *dma2*Δ cells more frequently than WT cells (Fig. 8d, e). These results imply that *dma1*Δ *dma2*Δ cells generate more ROS and are more susceptible to them compared to WT cells.

Yeast cells express two superoxide dismutases, Sod1 and Sod2, that detoxify the superoxide radical. To investigate genetic interactions between *DMA1/2* and *SOD1/2*, *SOD1* and *SOD2* genes were deleted in *dma1*Δ *dma2*Δ cells and cell growth was monitored. Compared to *dma1*Δ *dma2*Δ or *sod1*Δ *sod2*Δ double-mutant cells, *dma1*Δ *dma2*Δ *sod1*Δ *sod2*Δ quadruple mutant cells exhibited severe synthetic growth defects on a glucose-containing plate (Fig. 8f). Deletion of *SOD1*, but not of *SOD2*, was sufficient to cause the synthetic growth defect in *dma1*Δ *dma2*Δ cells, indicating that Sod1 mainly contributes to scavenging the superoxide radical in *dma1*Δ *dma2*Δ cells (Fig. 8g). Deletion of *SOD1* caused a severe growth defect in *mmr1*Δ cells expressing Mmr1-S414A mutant protein from its own promoter compared to that of *mmr1*Δ cells expressing unmutated Mmr1 (Fig. 8h), strongly suggesting that ROS toxicity observed in *dma1*Δ *dma2*Δ cells was a result of defective degradation of Mmr1.

## Discussion

**Fig. 9:**
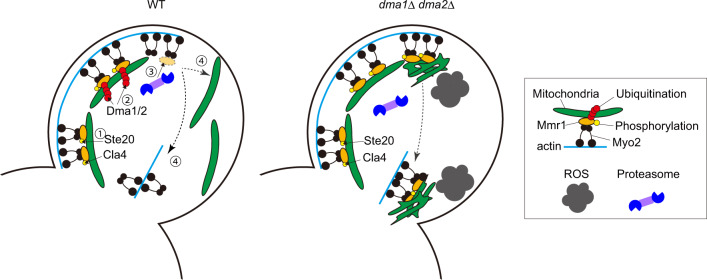
Model for release of mitochondria from myosin in the bud. In WT cells, mitochondria are transported to the growing bud by Myo2 on the actin cable. After mitochondria entered the growing bud, bud-localized kinases, Ste20 and Cla4, phosphorylate the adaptor protein Mmr1, most likely at S414 residue (step 1). Redundant E3 ligases, Dma1 and Dma2, recognize and poly-ubiquitinate the phosphorylated Mmr1 (step 2). Poly-ubiquitinated Mmr1 is degraded by the proteasome (step 3). Liberated mitochondria are widely distributed in the bud, while Myo2 moves to the bud neck (step 4). In *dma1*Δ *dma2*Δ cells, transported mitochondria are not released from Myo2, get stacked at the bud tip, and are deformed. Owing to the translocation of Myo2 to the bud neck, the mitochondria are transported to the bud neck and again get stacked there. Stacked and deformed mitochondria generate a higher level of ROS compared to normal mitochondria in WT cells.

In this research, we revealed that mitochondria are released from myosin through Dma1/2-mediated ubiquitination and subsequent degradation of an adaptor protein Mmr1 in *Saccharomyces cerevisiae* (Fig. 9). It was previously proposed that Mmr1 functions as a tether that anchors mitochondria to the bud tip^29^ alongside being an adaptor that bridges mitochondria and myosin. However, as observed in this study, the stacked mitochondria at the bud tip of *dma1*Δ *dma2*Δ cells, where Mmr1 is highly accumulated, did not remain there but translocated to the bud neck (Fig. 3, Supplementary Movie 2, and Supplementary Fig. 3). This suggests that the proposed role of Mmr1 as a tether is likely relatively minor or transient. Interestingly, Dma1/2 were previously reported to be involved in the correct positioning of the vacuole and peroxisome in the bud^23^. In the case of vacuole inheritance, Dma1/2 is proposed to ubiquitinate the adaptor protein, Vac17, leading to the release of the vacuole from the actin-myosin machinery. In this case, phosphorylation of Vac17 by Cla4 is proposed to be a critical step that triggers Vac17 ubiquitination by Dma1^30^. Vac17 turnover and vacuole release from Myo2 require additional factors, Yck3 and Vps41^31^. It is proposed that Yck3 and Vps41 form a complex and phosphorylate Vac17 to aid dissociation from Myo2. Both the Cla4-Dma1/2 axis and the Yck3-Vps41-dependent phosphorylation are required for Vac17 turnover. Therefore, we examined if Yck3 and Vps41 are also involved in Mmr1 turnover. We found that they are dispensable for the rapid turnover of Mmr1 (Supplementary Fig. 7). Considered together, although there exists a minor difference in regulatory factors required for release of the vacuole and mitochondria, it can be proposed that regulated degradation of adaptor proteins by the ubiquitin–proteasome system may be a general mechanism in yeast to release some organelles from the cytoskeleton for their correct positioning in the daughter cells. In mammalian cells, mitochondria positioning depends on microtubule and actin filaments^32,33^. It has been reported that symmetrical mitochondrial segregation during mammalian cell division requires action of the mitochondria myosin Myo19^34^. On the other hand, some reports describe that mitochondria segregation during cell division is passive, which does not require active transport of mitochondria on the cytoskeleton. However, even in that case, redistribution of mitochondria after cell division is dependent on cytoskeletal protein Cenp-F and mitochondria protein Miro^35^. Interestingly, before passive segregation of mitochondria during cell division, mitochondria motor proteins dissociate from mitochondria by the action of protein kinases CDK1 and Aurora A, leading to the release of mitochondria from microtubules^36^. This motor shedding is required for proper distribution and passive inheritance of mitochondria^36^. Identifying whether or not ubiquitin-mediated protein degradation of mitochondria motor proteins and/or adaptor proteins is involved in the regulation of mitochondrial dynamics before, during, and after cell division in mammalian cells would be an interesting topic for future research. Correct transport and positioning of melanosomes in mammalian cells also depend on the cytoskeleton. Melanosomes are transported in a polarized manner via motor proteins and adaptors^37^. Melanosomes are first directionally carried by motor protein kinesin on the microtubule cable, followed by switching over to the actin cable, and then transported directionally by myosin to be secreted out^37^. A series of such elaborate processes would certainly be spatiotemporally regulated. Therefore, it would also be of particular interest if the degradation of adaptor protein(s) plays an essential role in the directional transport and release of melanosomes from the cytoskeleton.

Interestingly, we often observed in WT cells that a part of tubular mitochondria in daughter cells, particularly around one of the two ends, was attached to Myo2 at the bud neck like an anchor or a fulcrum (Fig. 3e). This observation implies that a minor fraction of Mmr1 around one end of the transported mitochondria escapes degradation and maintains an association with Myo2 during cytokinesis. The mechanism and physiological significance of this phenomenon is another intriguing future research prospect.

Impairment of mitochondrial release from myosin caused their abnormal localization and morphology in *dma1*Δ *dma2*Δ cells. However, surprisingly, *dma1*Δ *dma2*Δ cells grew faster than WT cells on the plate containing glycerol, which suggested elevated respiratory activity in this mutant, while the growth of *dma1*Δ *dma2*Δ cells on the glucose-containing plate was slightly retarded (Fig. 7a). An increase in membrane potential-dependent incorporation of TMRM reagent and in cytochrome c levels in *dma1*Δ *dma2*Δ cells also supported increased respiratory activity in *dma1*Δ *dma2*Δ cells, in both media containing glucose or glycerol. The observed elevation in respiratory activity in *dma1*Δ *dma2*Δ cells was associated with an increase in ROS levels. Collectively, while increased respiratory activity positively impacts cell growth of *dma1*Δ *dma2*Δ cells in a glycerol-containing medium, it causes a mild growth defect in a glucose-containing medium. The negative impact of elevated ROS production may overwhelm the positive effect of effective ATP synthesis by respiration in a glucose-containing medium. However, the benefit of elevated respiratory activity probably surpasses the toxicity of ROS production in media containing non-fermentable carbon sources like glycerol since ATP synthesis in these media totally depends on respiration.

We suggested that Sod1 but not Sod2 mainly scavenges the superoxide radical in *dma1*Δ *dma2*Δ cells (Fig. 8g). Sod1 localizes to the cytosol and mitochondria, while Sod2 localizes only to mitochondria^38,39^. Therefore, both Sod1 and Sod2 can potentially scavenge the superoxide radicals in mitochondria. We speculate that Sod1 can function as a main superoxide dismutase in mitochondria due to its much higher, ~50-fold, expression level than Sod2^40^.

It has been reported that Dma1 and Dma2 are required to ensure the timely termination of telophase in glucose-containing medium^41^. Herein, we observed slight growth retardation of *dma1*Δ *dma2*Δ cells on the plate containing glucose (Fig. 7a). Interestingly, we observed the occasional mislocalization of Myo2 in the cytosol at the cytokinesis stage in *dma1*Δ *dma2*Δ cells, probably due to being caught up in the stacked mitochondria. It would be interesting to verify in future studies if the partial mislocalization of Myo2 in the cytokinesis stage accounts for, at least partly, the prolonged telophase and slight growth defect in *dma1*Δ *dma2*Δ cells. To minimize the potential pleiotropic effects of the double deletion of *DMA1* and *DMA2* on mitochondria dynamics and function, we studied the Mmr1-S414A variant; the results suggested that defects in mitochondrial dynamics and function observed in *dma1*Δ *dma2*Δ cells were accountable to a defective degradation of Mmr1 rather than their pleiotropic effects. Elucidation of the relationship between phosphorylation of the S414 residue of Mmr1, by Ste20 and Cla4, and the Dma1/2-mediated ubiquitination may further deepen our understanding of the mechanism of Mmr1 turnover. For this purpose, determination of the ubiquitination sites in Mmr1 and comparing them with those in the Mmr1-S414A variant, using both in vitro and in vivo assays, may be crucial to address in the future.

In this research, we discovered and analyzed the mechanism of regulated degradation of the mitochondria adaptor protein Mmr1. Spatiotemporally regulated degradation of Mmr1 was shown to be crucial for the proper distribution of mitochondria in daughter cells and for maintenance of mitochondrial homeostasis, although we cannot completely rule out the possibility that more mitochondria, including less-healthy ones, are inherited to the daughter cell in *dma1*Δ *dma2*Δ cells, and average mitochondria function was lowered in the daughter cell. These findings not only deepen our understanding of the mechanism and importance of adequate organelle inheritance during cell proliferation but also provide novel insights into the medical and pharmacological application to human diseases derived from dysregulation of organelle dynamics.

## Methods

### Yeast strains, media, and reagents

The *Saccharomyces cerevisiae* strains used in this work are listed in Supplementary Table 2. Cells were grown in YPD (2% d-glucose, 1% yeast extract, and 2% peptone) or synthetic medium (2% d-glucose and 0.67% yeast nitrogen base without amino acids) supplemented with appropriate amino acids, adenine, and uracil depending on the auxotrophy of the strain. In some experiments, as indicated in the text, 2% glycerol instead of glucose was used as the carbon source. For CHX chase experiments, a 5 mg/mL stock solution of cycloheximide (Nacalai Tesque, Kyoto, Japan, 06741-04) was prepared in H_2_O and directly added to the medium at a final concentration of 200 µg/mL. A 20 mM stock solution of MG132 (Peptide Institute, Osaka, Japan, 3175) was prepared in dimethylsulfoxide and was added to the medium at a final concentration of 50 µM. For the auxin-inducible degron system, a 500 mM stock solution of 3-indoleacetic acid (IAA; Nacalai Tesque, 19119-61), a typical auxin, was prepared in ethanol and added to the medium at a final concentration of 500 µM. A 20 µM stock solution of MitoProbe TMRM dissolved in dimethylsulfoxide was purchased from ThermoFisher Scientific (Waltham, USA, I34361) and added to the medium at a final concentration of 50 nM. A 2.5 mM CellROX Green reagent dissolved in dimethylsulfoxide was purchased from ThermoFisher Scientific (C10444) and used at a final concentration of 5 µM. A 14 µM stock solution of Acti-stain 488 Fluorescent phalloidin (Ctytoskelton, Denver, CO, PHDG1) was prepared in methanol and used at a final concentration of 1 µM.

### Genetic manipulation

Gene disruption was performed by replacing the entire coding sequence of the genes with a marker gene via homologous recombination. Chromosome fusions of *HA*, *HA-AID*, or *GFP* to the 3ʹ-terminus of the gene were conducted using PCR-based gene disruption and modification methods^42^. Briefly, the sequence containing the tag-encoding gene (*HA*, *HA-AID*, or *GFP*), the *ADH1* terminator, and a marker gene was amplified using PCR from pFU562 (for HA-tagging), pOK521 (for HA-AID-tagging)^43^, or pOK617 (for GFP-tagging; this study) with a primer set containing the homologous region of each gene. The PCR-amplified fragments were directly inserted into the chromosome via homologous recombination. Successful deletions of the genes and tagging were confirmed via genomic PCR, immunoblot, and/or fluorescence microscopy. The sequence encoding Mt-GFP was integrated into the *URA3* or *LEU2* locus as follows. The *GPD* promoter sequence followed by *Mt-GFP* sequence and the *CYC1* terminator was excised from p416-GPD-mtGFP (kind gift from Dr. K. Okamoto, Osaka University) by digestion with *Sac*I and *Eag*I and cloned into the *Sac*I-*Eag*I site of pRS306 or pRS305^44^. The resultant plasmids were linearized by digestion with *Stu*I or *Afl*II and integrated into *URA3* or *LEU2* locus, respectively, via homologous recombination. Mt-mCherry strains were constructed by the same method except that the starting material was p416-GPD-mtCherry (kind gift from Dr. K. Okamoto, Osaka University).

*S. cerevisiae* Ubc4 was subcloned into pRSET B vector (Invitrogen, Carlsbad, USA) with an amino-terminal His_6_ tag. *S. cerevisiae* Mmr1 containing amino-terminal His_6_ and 3 × HA tags, and *S. cerevisiae* Ste20 containing amino-terminal His_6_ and FLAG tags were subcloned into pFastBacHTc vector (Invitrogen). *S. cerevisiae* Cla4 containing amino-terminal His_6_ and FLAG tags was subcloned into the pFastBacHTa vector (Invitrogen). Expression vectors for mouse ubiquitin and *S. cerevisiae* Uba1 have been described previously^45^.

### Co-immunoprecipitation

Cells were grown to log phase in YPD medium at 30 °C, further cultured for 3 h at 37 °C, and collected. Cells were resuspended in TBS containing EDTA-free protease inhibitor cocktail (Complete, Roche Diagnostics, Indianapolis, USA, 11836170001), and broken by vigorously mixing with glass beads. After transferring the lysate to a new tube without the glass beads, the proteins were solubilized with 1% Triton X-100 for 1 h at 4 °C. Debris and unsolubilized heavy structures were removed by centrifugation at 20,000 × *g* for 5 min at 4 °C. After the supernatants were transferred to a new tube, samples were centrifuged again at 20,000 × *g* for 10 min at 4 °C to remove debris and unsolubilized heavy structures. A portion of the supernatants was pooled as input fractions. The remaining solubilized fraction was incubated with anti-HA antibody (1:200 dilution; 12CA5; Sigma, St. Louis, USA, 11666606001) for 40 min at 4 °C while rotating. After adding Protein A Sepharose (GE Healthcare Biosciences, Piscataway, USA, 17528004), samples were maintained for 15 min at 4 °C while rotating. Beads were collected by centrifugation at 1500 × *g* for 1 min at 4 °C, and the supernatant was removed. After washing the thrice with TBS containing 0.1% Tween-20, proteins were eluted by incubation in SDS-sample buffer for 5 min at 88 °C and subjected to immunoblot analysis.

### Immunoblot analysis

Cell lysates were prepared using the alkaline-trichloroacetic acid method as described previously^46^. Briefly, harvested cells were resuspended in an ice-cold solution containing 0.25 N NaOH and 1% (v/v) 2-mercaptoethanol and incubated on ice for 10 min. Trichloroacetic acid was added at a final concentration of 7% (w/v), and the samples were incubated on ice for 10 min. After centrifugation at 20,000 × *g* for 2 min at 4 °C, the supernatant was removed. Then, 1 M tris without pH adjustment was gently added, ensuring to not break the pellet, to neutralize the remaining trichloroacetic acid, and centrifuged at 20,000 × *g* for 30 s. The supernatant was removed, and proteins were eluted by incubation in SDS-sample buffer for 10 min at 37 °C. After centrifugation at 10,000 × *g* for 1 min at room temperature, the supernatant was transferred to a new tube and subjected to immunoblotting. Proteins were separated via SDS-PAGE and transferred to an Immobilon^TM^ polyvinylidene difluoride membrane (Millipore, Billerica, USA). The membrane was incubated with anti-Mmr1 (1:200 dilution; our laboratory), anti-Pgk1 (1:3000 dilution; 22C5D8, ThermoFisher Scientific, UA2696317), anti-Cdc48 (1:10,000 dilution; our laboratory), anti-HA (1:2000 dilution; TANA2, Medical and Biological Laboratories, M180-3), anti-Myc (1:5000 dilution; our laboratory), anti-Tom70 (1:4000 dilution; gift from Dr. Y. Tamura, Yamagata University), anti-Tim23 (1:2000 dilution; gift from Dr. Y. Tamura, Yamagata University), anti-Cyc1 (1:2000 dilution; gift from Dr. Y. Tamura, Yamagata University), anti-Cyb2 (1:2000 dilution; gift from Dr. Y. Tamura, Yamagata University), or anti-Hsp60 (1:4000 dilution; gift from Dr. Y. Tamura, Yamagata University) antibody. HRP-conjugated anti-mouse IgG (1:7500 dilution; Sigma, A4416) or HRP-conjugated anti-rabbit IgG (1:7500 dilution; Sigma, A6154) was used as the secondary antibody. Immunodetection was performed using a Luminata Forte Western HRP Substrate system (Merck Millipore, Burlington, USA, 61-0206-81) or a Chemi-Lumi One L system (Nacalai Tesque, 07880) with a bioanalyzer (LAS4000 mini; GE healthcare Biosciences) or with X-ray films.

### Immunoprecipitation of Mmr1-HA from yeast cells

Cells expressing Mmr1-HA were grown to log phase at 25 °C, further cultured for 2 h at 37 °C, and harvested. Cells were broken by vigorously mixing with glass beads in a solution containing 40 mM Tris–HCl (pH 7.5), 150 mM NaCl, 1 mM dithiothreitol, 0.5% Triton X-100, EDTA-free protease inhibitor cocktail (Complete, Roche, 11836170001), and EDTA-free Phosphatase Inhibitor Cocktail (Nacalai Tesque, 07575-51). After removing the debris and glass beads by centrifugation at 20,000 × *g* for 5 min, lysates were incubated with anti-HA (1:200 dilution; 12CA5; Sigma, 11666606001) antibody and Dynabeads Protein G (Invitrogen, 100-03D) for 30 min at 4 °C. The beads were then washed thrice with a solution containing 40 mM Tris–HCl (pH 7.5), 150 mM NaCl, 1 mM dithiothreitol, and 0.5% Triton X-100. Immunoprecipitated proteins were partially separated (~1 cm) via SDS-PAGE. The gel lane for each condition was cut horizontally into several pieces (~0.2 cm^3^ for each piece) and subjected to protein mass spectrometric analysis.

### Protein mass spectrometry

In-gel digestion was performed according to the method described by Shevchenko and colleagues^47^. Samples were analyzed using nano-flow reverse-phase liquid chromatography followed by tandem MS. A capillary reverse-phase HPLC-MS/MS system was composed of a Dionex U3000 gradient pump equipped with a VICI Cheminert valve and Q Exactive equipped with a nano-electrospray ionization (NSI) source (AMR, Tokyo, Japan). The desalted peptides were loaded into a separation capillary C18 reverse-phase column (NTCC-360/100-3-125, 125 × 0.1 mm, Nikkyo Technos, Tokyo, Japan). Xcalibur 3.0.63 system (Thermo) was used to record peptide spectra over the mass range of *m/z* 350–1800. Repeatedly, MS spectra were recorded, followed by ten data-dependent high energy collisional dissociation (HCD) MS/MS spectra generated from the ten highest intensity precursor ions. MS/MS spectra were interpreted, and peak lists were generated using Proteome Discoverer 2.2.0.388 (Thermo). Searches were performed using the SEQUEST (Thermo) program against *Saccharomyces cerevisiae* (SwissProt TaxID = 559292) peptide sequence. Search parameters were set as follows: enzyme selected as used with two maximum missing cleavage sites, a mass tolerance of 10 ppm for peptide tolerance, 0.02 Da for MS/MS tolerance, fixed modification of carbamidomethyl (C), and variable modification of oxidation (M), phosphorylation (S, T, Y), and ubiquitination (K). Peptide identifications were based on significant Xcorr (high confidence filter). Peptide identification and modification data returned from SEQUEST were manually inspected and filtered to obtain confirmed peptide identification and modification lists of HCD MS/MS.

### In vivo ubiquitination assay

Cells were grown to log phase in YPD medium at 25 °C, further cultured for 90 min at 37 °C, and harvested. Cells were resuspended in lysis buffer [40 mM HEPES–NaOH (pH 7.5), 8 M urea, 50 mM NaCl, and 20 mM imidazole] and broken by vigorously mixing with glass beads. Lysates were centrifuged at 20,000 × *g* for 10 min at 4 °C to remove debris and glass beads. Supernatants were transferred to a new tube, and a portion of the sample was pooled as the input fraction and was also used to measure the protein concentration. Lysates with the same total protein amounts were subjected to Ni^2+^pull-down using Ni^2+^-agarose (FUJIFILM Wako Pure Chemical, Osaka, Japan, 149-07984) for 90 min at 25 °C. Beads were washed four times with wash buffer [40 mM HEPES–NaOH (pH 7.5), 4 M urea, 50 mM NaCl, and 20 mM imidazole]. After removing the wash buffer, beads were incubated in SDS-sample buffer for 5 min at 88 °C to elute proteins.

### In vitro phosphorylation and ubiquitination assays

Myc-Uba1-His_6_, His_6_-Ubc4, and His_6_-ubiquitin were expressed in *E. coli* BL21 (DE3) and purified using Ni^2+^-agarose affinity chromatography (FUJIFILM Wako Pure Chemical, 149-07984). His_6_-FLAG-Ste20, His_6_-FLAG-Cla4, and His_6_-FLAG-Dma1 were purified using Ni^2+^-agarose chromatography from lysates of Sf21 insect cells (ThermoFisher Scientific, B82101) infected with baculoviruses encoding the tagged Ste20, Cla4, and Dma1. The supernatants of Sf21 cells infected with baculoviruses encoding His_6_-3 × HA-Mmr1 were immunoprecipitated with anti-HA (1:200 dilution; 12CA5; Sigma, 11666606001) antibody and protein A Sepharose beads (GE Healthcare Biosciences, 17528004). For the in vitro Mmr1 phosphorylation assay, the beads binding the tagged Mmr1 were incubated with 200 ng of His_6_-FLAG-Ste20 or His_6_-FLAG-Cla4 in a 50 µL solution containing 40 mM Tris–HCl (pH 7.5), 60 mM NaCl, 1 mM dithiothreitol, 5 mM MgCl_2_, 10% (v/v) glycerol, and 1.5 mM ATP at 30 °C for 1 h. For the in vitro Mmr1 ubiquitination assay, beads binding the tagged Mmr1 were washed twice with a solution containing 40 mM Tris–HCl (pH 7.5), 60 mM NaCl, 1 mM dithiothreitol, 5 mM MgCl_2_, 10% (v/v) glycerol, and 1.5 mM ATP after the in vitro phosphorylation reaction, and then incubated with 30 ng of Myc-Uba1-His_6_, 30 ng of His_6_-Ubc4, and 2 μg of His_6_-ubiquitin in a 20 µL solution containing 40 mM Tris–HCl (pH 7.5), 60 mM NaCl, 1 mM dithiothreitol, 5 mM MgCl_2_, 10% (v/v) glycerol, and 1.5 mM ATP. Reaction mixtures were incubated at 30 °C for the periods indicated in Fig. 5.

### Fluorescence microscopy

Cells were grown to log phase in synthetic glucose or synthetic glycerol medium and observed under a fluorescence microscope (AxioObserver Z1; Carl Zeiss, Oberkochen, Germany) equipped with a CCD camera (AxioCam MRm; Carl Zeiss). To quantify the stacked mitochondria, cells with large buds were selected for the analysis. For time-lapse imaging of mitochondria, cells were cultured in a glass-based culture dish (Iwaki, Chiba, Japan) and observed at room temperature. Images were processed using Photoshop CS3 (Adobe, San Jose, USA). Polymerized form of actin (F-actin) was visualized as follows. Cells were grown to log phase in YPD medium at 30 °C. Cells were fixed by adding formaldehyde directly to the medium at a final working concentration of 5%, followed by incubation for 30 min at 30 °C. Cells were collected by centrifugation, resuspended in PBS containing 5% formaldehyde, and incubated for 30 min at 30 °C. After washing with PBS, cells were resuspended in PBS containing 0.1% (v/v) Triton X-100 and incubated for 5 min at room temperature. Cells were washed with PBS and resuspended in PBS containing 1 µM Acti-stain 488 Fluorescent phalloidin (Ctytoskelton, PHDG1) and incubated in the dark for 1 h at room temperature. Cells were washed with PBS, resuspended in PBS, and subjected to microscopy. For 3D imaging of mitochondria, growing cells were fixed by adding formaldehyde at a final working concentration of 5% for 30 min at 30 °C. Cells were collected by centrifugation, resuspended in PBS containing 5% formaldehyde, and incubated for 20 min at room temperature. Cells were then washed with PBS and observed under an all-in-one fluorescence microscope (BZ-X800; Keyence, Osaka, Japan) equipped with a structured-illumination microscopy system. Each optical section was obtained at an interval of 0.2 µm, deconvoluted, and reconstructed into a 3D image. Cells stained with MitoProbe TMRM were observed under an all-in-one fluorescence microscope (BZ-X810; Keyence). For quantification of the fluorescence signal of TMRM and CellROX green reagents, more than 100 cells were analyzed using the Image J software.

### Electron microscopy

Cells in the log phase were sandwiched between copper disks and frozen in liquid propane. The frozen cells were then freeze-substituted with acetone containing 2% glutaraldehyde and 2% tannic acid. After washing with acetone, samples were further fixed with 2% osmium tetroxide in acetone and dehydrated with ethanol. Samples were then infiltrated with propylene oxide twice and with a 70:30 mixture of propylene oxide and resin (Quetol-651; Nisshin EM, Tokyo, Japan), and the propylene oxide was volatilized. Samples were transferred to fresh 100% resin, and the resin was polymerized at 60 °C. Ultrathin sections (70 nm thick) of the blocks were prepared with an ultramicrotome (Ultracut UCT, Leica Microsystems, Wetzlar Germany). The sections were placed on copper grids, stained with lead stain solution (Sigma), and observed under a transmission electron microscope (JEM-1400Plus, JOEL, Tokyo, Japan).

### Reporting summary

Further information on research design is available in the Nature Research Reporting Summary linked to this article.

## Supplementary information

Supplementary Information

Description of Additional Supplementary Files

Supplementary Data 1

Supplementary Movie 1

Supplementary Movie 2

Supplementary Movie 3

Supplementary Movie 4

Supplementary Movie 5

Reporting Summary

## Data Availability

The LC-MS/MS data generated in this study have been deposited in the jPOST database under accession code PXD032866. Source data are provided with this paper.

## Source data

Source Data
