# Supplementary material for: Proteolysis of adaptor protein Mmr1 during budding is necessary for mitochondrial homeostasis in Saccharomyces cerevisiae
Source: Nat Commun. 2022 Apr 14;13:2005. doi: 10.1038/s41467-022-29704-8 (PMC9010424; doi:10.1038/s41467-022-29704-8)
Supplement: Supplementary file 1 — Supplementary Information [file 41467_2022_29704_MOESM1_ESM.pdf]

Supplementary Information

Proteolysis of adaptor protein Mmr1 during budding is necessary for mitochondrial homeostasis in *Saccharomyces cerevisiae*

Keisuke Obara, Taku Yoshikawa, Ryu Yamaguchi, Keiko Kuwata, Kunio Nakatsukasa, Kohei Nishimura, and Takumi Kamura

Supplementary Table 1 | E3 ubiquitin ligases co-purified with Mmr1-HA

Proteins annotated as E3 ubiquitin ligase were extracted using data from Supplementary Data 1 and listed. Detected peptides are also listed.

Accession	Description	ORF	Detected peptide
P38823	E3 ubiquitin-protein ligase DMA1 [OS=Saccharomyces cerevisiae S288C]	YHR115C	[K].SSSGTFLNHQR.[L]
			[R].LTPFIDTSSTSVANQGLFFDPIIR.[T]
P53924	E3 ubiquitin-protein ligase DMA2 [OS=Saccharomyces cerevisiae S288C]	YNL116W	[R].NIVGGADGSTIVNNSQEMYK.[N]
			[K].SSSGTFLNHQR.[L]
			[K].LTTGIEEEDCSICLCK.[I]
			[K].AGPGSQLVIGR.[Y]
			[K].IPEQYHPVVFK.[S]
			[R].DGDILQLGMDFR.[G]
			[R].LTPFIDSSSTTNQGLFFEPIIR.[K]
P19812	E3 ubiquitin-protein ligase UBR1 [OS=Saccharomyces cerevisiae S288C]	YGR184C	[R].QGVPDNVHIDLLTSR.[I]
			[K].LSNPELTVFPDSLEDAVDIDK.[I]
			[K].ILPESSTHSLSPLINDVETPTSR.[T]
			[R].LQHILYFDNR.[Y]
			[R].NGMSVLHQASYYK.[N]
			[K].TLCSEYLNATECR.[D]
			[K].ITSQQTIPFYK.[I]
			[K].SDVFSESIVR.[K]
			[R].LFKPFTEENYR.[T]
			[K].EIISLLTLCK.[L]
			[R].QGVNLQETEK.[E]
			[K].LFLNAIR.[I]
			[R].YIDLSILADGNQIPLGHHK.[I]
			[R].SIHNLPYFR.[Y]
			[K].ICLTCGVK.[V]
			[K].NNPELGSYSR.[D]
			[K].ISDFSLR.[S]
			[K].SYSISFK.[Q]
P12868	E3 ubiquitin-protein ligase pep5 [OS=Saccharomyces cerevisiae S288C]	YMR231W	[K].VLRPIIEGER.[M]
P24814	SCF E3 ubiquitin ligase complex F-box protein GRR1 [OS=Saccharomyces cerevisiae S288C]	YJR090C	[R].LIDLSGCENITDK.[T]
			[R].ITDASLFQLSK.[L]
			[R].IQYVDFACCTNLTNR.[T]
			[R].TLYELADLPK.[L]
			[R].ALETENMEIR.[N]
			[K].ITANNNMNDELVELLANK.[C]
			[R].MSSEEVNSLLLQVNK.[T]
			[R].EINSEMCHIVR.[K]
			[K].FLQSVDITGIR.[D]
			[R].QIFCVFSGK.[G]
			[K].SQLDLFLR.[T]
			[K].SLYAYCHSK.[L]
			[K].VVDDMPSLR.[L]
			[R].VQGFYVPQAR.[N]
			[K].TFCEDPFSDVDDDQDYVVAPGVNR.[E]
			[R].KFHELNDHIDDFEVNVASLVR.[V]
			[R].DVSDDVFDTLATYCPR.[V]
			[R].NFIVHSPMLK.[R]
P39940	E3 ubiquitin-protein ligase RSP5 [OS=Saccharomyces cerevisiae S288C]	YER125W	[R].FGEVVTVDLKPDGR.[N]
Q07963	E3 ubiquitin-protein ligase UBR2 [OS=Saccharomyces cerevisiae S288C]	YLR024C	[K].FTSCEPVLR.[A]
P53119	Probable E3 ubiquitin-protein ligase HUL5 [OS=Saccharomyces cerevisiae S288C]	YGL141W	[K].LSHNFLSSYPNSLGNR.[Q]
			[K].LLNMTTDEIK.[S]
			[R].DNVLEDAFNAFNSIGER.[F]
			[R].ASFLSYSSASK.[L]
			[K].SLDLTFEIDEPESSAK.[V]
			[K].FVTSVPQAPLQGFK.[A]

Supplementary Table 2 | Yeast Strains used in this study.

Name	Genotype	Source
W303-1a	*MAT*a *ade2-1 ura3-1 his3-11,15 trp1-1 leu2-3,112 can1-100*	Y. Kikuchi
JB291	*MAT*a *ura3 his3-11,15 leu2-3,112*	[1, 2]
JB290	JB291, *pre1-1 pre2-1*	[1, 2]
BY4741	*MATa his3*Δ1 *leu2*Δ *ura3*Δ *met15*Δ	[3]
YTK4652	W303-1a, *pdr5*Δ::*HPT*	This study
YPH499	*MATa his3*Δ*200 lys2-801 ade2-101 leu2*Δ1 *ura3-52 trp1*Δ63	[4]
CMY253	*MAT*a *ade2-101 ura3-52 his3*Δ*200 leu2*Δ*1 lys2-801*	[5]
CMY762	CMY253, *cim3-1*	[5]
YTK3487	CMY762, *MMR1-3HA::LEU2*	This study
YTK5423	CMY762, *DMA1-5FLAG*::*HIS3*	This study
YTK5425	CMY762, *DMA1-5FLAG*::*HIS3 MMR1-3HA::LEU2*	This study
YTK6168	CMY762, *His-Myc-Ubiquitin*::*URA3*	This study
YTK5810	CMY762, *MMR1-3HA::LEU2 His-Myc-Ubiquitin::URA3*	This study
YTK5893	CMY762, *MMR1-3HA::LEU2 dma1*Δ*::KanMX4 dma2*Δ::*HISMX6 His-Myc-ubiquitin::URA3*	This study
YTK5414	W303-1a, *dma1*Δ::*KanMX4*	This study
YTK5415	W303-1a, *dma2*Δ::*HISMX6*	This study
YTK5416	W303-1a, *dma1*Δ::*KanMX4 dma2*Δ::*HISMX6*	This study
YTK5334	W303-1a, P_{GPD}-*Mt-GFP*-T_{CYC1}::*URA3*	This study
YTK5341	W303-1a, P_{GPD}-*Mt-GFP*-T_{CYC1}::*URA3 dma1*Δ::*KanMX4 dma2*Δ::*HISMX6*	This study
YOK5303	W303-1a, P_{GPD}-*Mt-mCherry*-T_{CYC1}::*URA3 MYO2-GFP*::*LEU2*	This study
YOK5304	W303-1a, P_{GPD}-*Mt-mCherry*-T_{CYC1}::*URA3 MYO2-GFP*::*LEU2 dma1*Δ::*KanMX4 dma2*Δ::*HISMX6*	This study
YOK5182	W303-1a, P_{GPD}-*Mt-mCherry*-T_{CYC1}::*URA3*	This study
YOK5185	W303-1a, P_{GPD}-*Mt-mCherry*-T_{CYC1}::*URA3 dma1*Δ::*KanMX4 dma2*Δ::*HISMX6*	This study
YOK5595	W303-1a, P_{GPD}-*Mt-mCherry*-T_{CYC1}::*URA3 MMR1-GFP*::*ADE2*	This study
YOK5596	W303-1a, P_{GPD}-*Mt-mCherry*-T_{CYC1}::*URA3 MMR1-GFP*::*ADE2 dma1*Δ::*KanMX4 dma2*Δ::*HISMX6*	This study
YTK5879	W303-1a, *ste20*Δ::*KanMX4*	This study
YOK5305	W303-1a, *STE20-GFP*::*LEU2*	This study
YOK5407	W303-1a, *CLA4-GFP*::*LEU2*	This study
YTK5433	W303-1a, *mdm12*Δ::*NatNT2*	This study
BY25598	W303-1a, P_{ADH1}-*OsTIR1-9Myc*::*URA3*	This study
YOK5411	BY25598, *CLA4-3HA-AID::KanMX6*	This study
YOK5408	BY25598, *ste20*Δ::*NatNT2*	This study
YOK5409	BY25598, *ste20*Δ::*NatNT2 CLA4-3HA-AID*::*KanMX6*	This study
YOK5434	BY25598, *ste20*Δ::*NatNT2 CLA4-3HA-AID*::*KanMX6* P_{GPD}-*Mt-GFP*-T_{CYC1}::*LEU2*	This study
YTK5777	W303-1a, *mmr1*Δ::*KanMX4 MMR1::URA3*	This study
YTK5778	W303-1a, *mmr1*Δ::*KanMX4 MMR1(S414A)::URA3*	This study
YTK5984	W303-1a, *mmr1*Δ::*KanMX4 MMR1(S266A)::URA3*	This study
YTK6021	W303-1a, *mmr1*Δ::*KanMX4 MMR1(S155A)::URA3*	This study
YTK6316	W303-1a, *mmr1*Δ::*KanMX4 MMR1(S76A)::URA3*	This study
YOK5455	W303-1a, P_{GPD}-*Mt-GFP*-T_{CYC1}::*LEU2 mmr1*Δ::*KanMX4 MMR1::URA3*	This study
YOK5456	W303-1a, P_{GPD}-*Mt-GFP*-T_{CYC1}::*LEU2 mmr1*Δ::*KanMX4 MMR1(S414A)::URA3*	This study
YOK5464	W303-1a, *sod1*Δ::*KanMX4*	This study
YOK5466	W303-1a, *sod2*Δ::*LEU2*	This study

YOK5468	W303-1a, *sod1* Δ::*KanMX4 sod2* Δ::*LEU2*	This study
YOK5479	W303-1a, *dma1* Δ::*NatNT2 dma2* Δ::*HISMX6 sod1* Δ::*KanMX4*	This study
YOK5480	W303-1a, *dma1* Δ::*NatNT2 dma2* Δ::*HISMX6 sod2* Δ::*LEU2*	This study
YOK5481	W303-1a, *dma1* Δ::*NatNT2 dma2* Δ::*HISMX6 sod1* Δ::*KanMX4 sod2* Δ::*LEU2*	This study
YOK5482	W303-1a, *mmr1* Δ::*NatNT2 MMR1::URA3*	This study
YOK5483	W303-1a, *mmr1* Δ::*NatNT2 MMR1(S414A)::URA3*	This study
YOK5485	W303-1a, *mmr1* Δ::*NatNT2 MMR1::URA3 sod1* Δ::*KanMX4*	This study
YOK5486	W303-1a, *mmr1* Δ::*NatNT2 MMR1(S414A)::URA3 sod1* Δ::*KanMX4*	This study
YOK5261	BY4741, *dma1* Δ::*NatNT2 dma2* Δ::*HIS3*	This study
4814	BY4741, *ubr1* Δ::*KanMX4*	[6]
YOK5581	BY4741, *pep5* Δ::*KanMX4*	This study
6902	BY4741, *grr1* Δ::*KanMX4*	[6]
1579	BY4741, *ubr2* Δ::*KanMX4*	[6]
4508	BY4741, *hul5* Δ::*KanMX4*	[6]
6122	BY4741, *yck3* Δ::*KanMX4*	[6]
4015	BY4741, *vps41* Δ::*KanMX4*	[6]
FAY51R	YPH499, *rsp5-1*	[7]

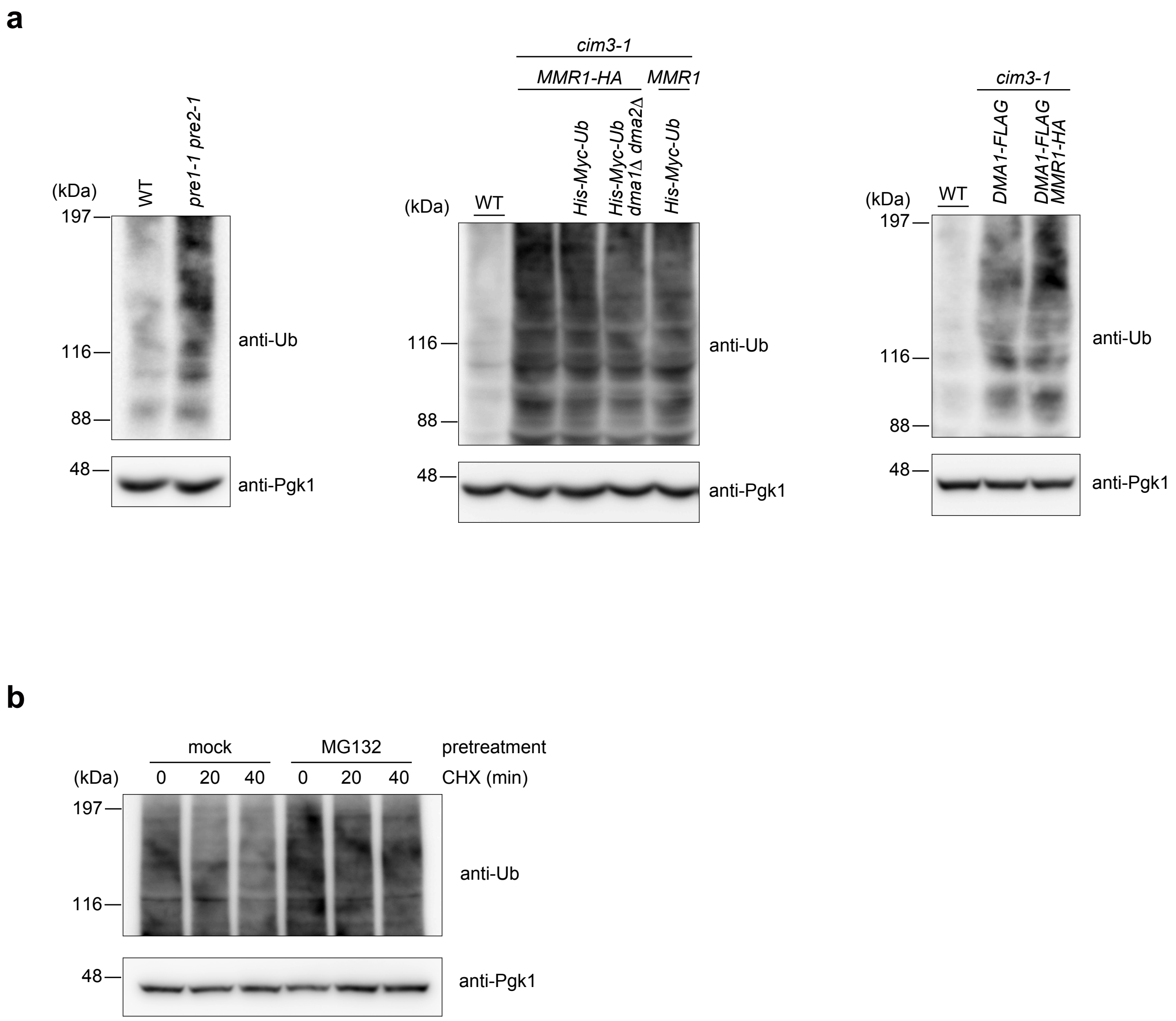

Supplementary Fig. 1 | Ubiquitinated proteins are accumulated in proteasome mutants and cells treated with MG132

a) JB291 (WT), JB290 (*pre1-1 pre2-1*), CMY253 (WT), YTK3487 (*cim3-1 MMR1-HA*), YTK5810 (*cim3-1 MMR1-HA His-Myc-Ub*), YTK5893 (*cim3-1 MMR1-HA His-Myc-Ub dma1*Δ *dma2*Δ), YTK6168 (*cim3-1 His-Myc-Ub*), YTK5423 (*cim3-1 DMA1-FLAG*), and YTK5425 (*cim3-1 DMA1-FLAG MMR1-HA*) cells were grown to log phase in YPD medium at permissive temperature (25˚C), further incubated for 2 h at the restrictive temperature (37˚C), and harvested. Their total lysates were subjected to immunoblot analysis with anti-ubiquitin (Ub) or, to demonstrate uniform loading, anti-Pgk1 antibody. b) YTK4652 (*pdr5*Δ) cells were grown to log phase in YPD medium at 30˚C, treated with MG132 or mock-treated with dimethyl sulfoxide for 40 min, treated with cycloheximide (CHX), and harvested. Their total lysates were subjected to immunoblot analysis with anti-Ub or anti-Pgk1 antibodies. Similar results were obtained from two independent experiments.

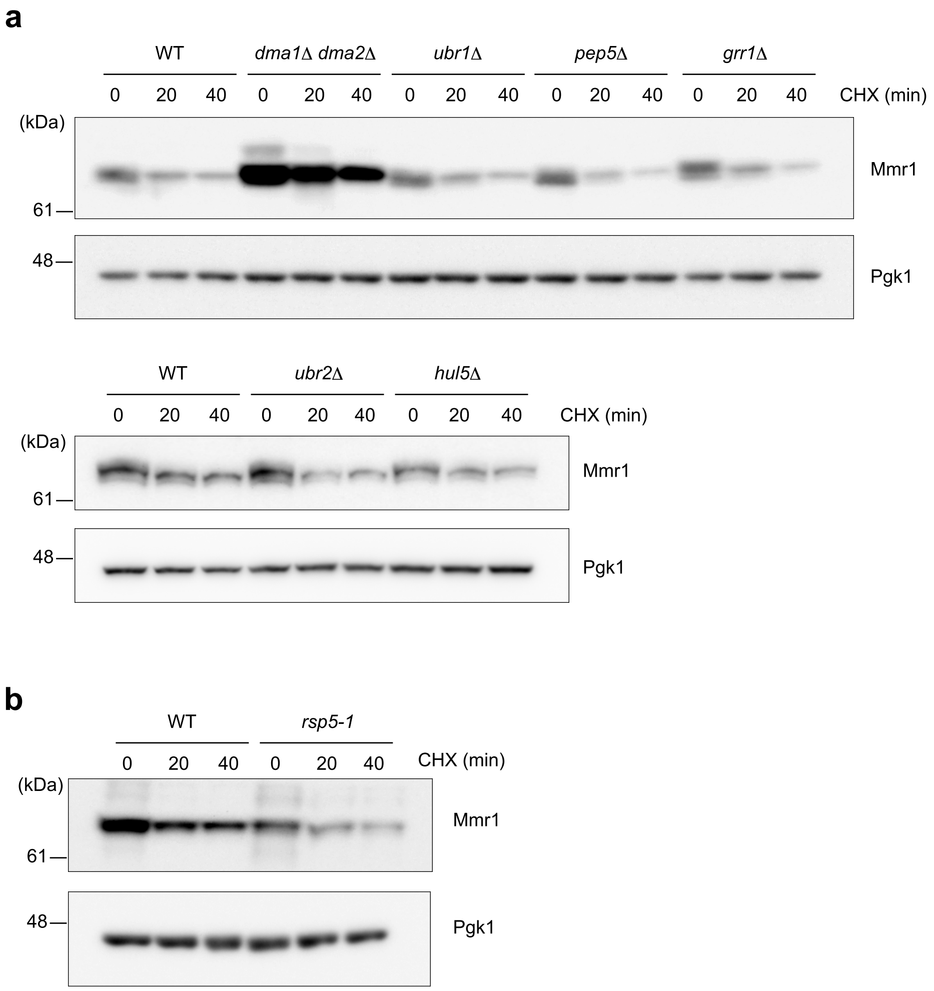

Supplementary Fig. 2 | Dma1 and Dma2 are involved in Mmr1 turnover

a) BY4741 (WT), YOK5261 (*dma1*Δ *dma2*Δ), 4814 (*ubr1*Δ), YOK5581 (*pep5*Δ), 6902 (*grr1*Δ), 1579 (*ubr2*Δ), and 4508 (*hul5*Δ) cells grown to log phase in YPD medium at 30˚C were treated with cycloheximide (CHX) and harvested. Their total lysates were prepared and subjected to immunoblot analysis with anti-Mmr1 or, to demonstrate uniform loading, anti-Pgk1 antibody. b) YPH499 (WT) and FAY51R (*rsp5-1*) cells grown to early log phase in YPD medium at 25˚C were further cultured for 2 h at 37˚C, and subjected to CHX-chase assay. Mmr1 and Pgk1 were detected via immunoblotting as in a. Similar results were obtained from two independent experiments.

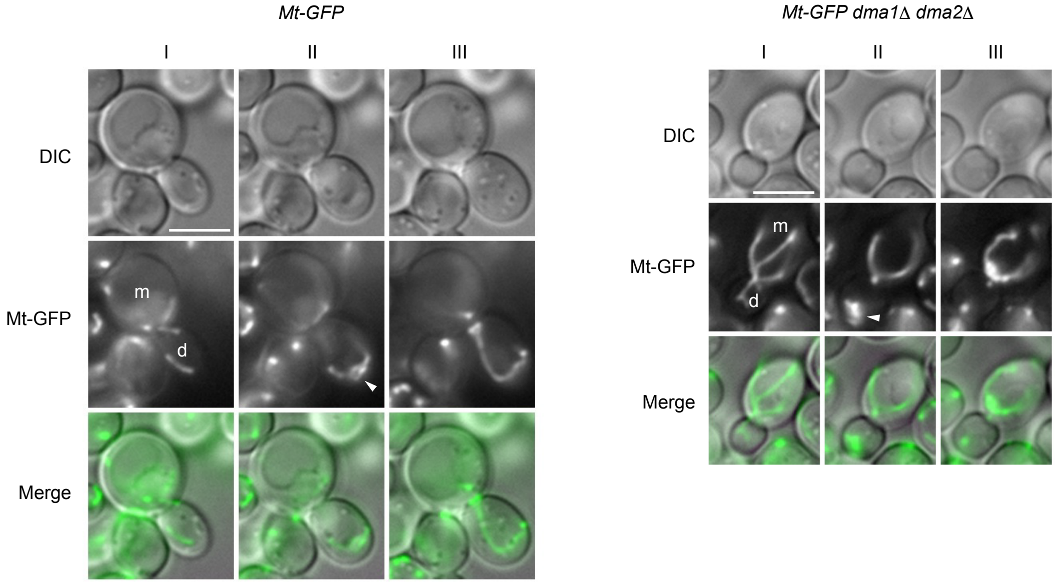

Supplementary Fig. 3 | Mitochondria dynamics during budding

YTK5334 (*Mt-GFP*) and YTK5341 (*Mt-GFP dma1*Δ *dma2*Δ) cells were grown to log phase in synthetic complete medium and serially observed under a fluorescence microscope using glass-base dishes. Representative images are shown. Cells with mitochondria being transported into the growing bud (I), stacked temporally at the bud-tip (II; arrowhead), and moving dynamically in *Mt-GFP* daughter cells or pulled back to the bud-neck and stacked in *Mt-GFP dma1*Δ *dma2*Δ cells (III) are shown. For movies of *Mt-GFP* and *Mt-GFP dma1*Δ *dma2*Δ cells, please see Supplementary Movie 1 and 2, respectively. m and d, mother cell and daughter cell, respectively. DIC, Differential interference contrast. Bar, 5 µm.

a

H	Master I	Q06324	Mitochondrial MYO2 receptor-related protein 1 [OS=Saccharomyces cerevisiae S288C]	
C	Confide	Annotated Sequence	Modifications	
	High	[K].KLTESSAVVATSTSK.[T]		
	High	[K].TTSEGQNSNPDPLNIEK.[N]		
	High	[K].LDTGPIGNSLLYPTSLSK.[L]		
	High	[R].FMLEVMKDPSIQAQR.[S]	1xOxidation [M2(100)]	
	High	[R].FMLEVMKDPSIQAQR.[S]		
	High	[R].GFQLNLPVQVEK.[K]		
	High	[K].RGFQLNLPVQVEK.[K]		
	High	[R].NAGSFQNLLNSPTK.[L]		
	High	[K].NAEGLLPSYVPVPATPLEDPENHGVR.[K]		
	High	[K].MLKPEYLSQTTSNLPLLSALLK.[N]		
	High	[K].NAEGLLPSYVPVPATPLEDPENHGVRK.[V]		
	High	[K].LTESSAVVATSTSK.[T]		
	High	[K].DLNLDNLPTDNNGFVQYGLKGNNNNNR.[Y]	1xGG [K20(100)]	
	High	[K].DLNLDNLPTDNNGFVQYGLK.[G]		
	High	[R].VVSGGSTQCLSTEVNELPK.[D]	1xCarbamidomethyl [C9]	
	High	[R].VVSGGSTQCLSTEVNELPK.[D]	1xCarbamidomethyl [C9]; 1xPhospho [S6(99.6)]	S224
	High	[R].SSESVAQVQK.[K]		
	High	[R].YSFISSTSTDYEPEWCDGQQHISMQMASMANAEEANSR.[E]	1xCarbamidomethyl [C16]	
	High	[K].QLELEITELK.[L]		
	High	[K].LVHSMTTNR.[Y]		
	High	[K].ERNPPSFEAK.[V]		
	High	[K].VCAEEPILRK.[N]	1xCarbamidomethyl [C2]	
	High	[R].FQFLNNTPK.[M]		
	High	[K].VCAEEPILR.[K]	1xCarbamidomethyl [C2]	
	High	[K].KTTSEGQNSNPDPLNIEK.[N]		
	High	[K].LVHSMTTNR.[Y]	1xOxidation [M5(100)]	
	High	[R].FMLEVMK.[D]	1xOxidation [M2(100)]	
	High	[K].DPSIQAQR.[S]		
	High	[R].VSVLDLK.[K]		
	High	[K].IKQLELEITELK.[L]	1xGG [K2(100)]	
	High	[K].TEGNSAR.[I]		
	High	[R].FMLEVMK.[D]		
	High	[R].LARVSVLDLKK.[I]	1xPhospho [S5(100)]	S414
	High	[K].MLKPEYLSQTTSNLPLLSALLK.[N]	1xOxidation [M1(100)]	
	High	[R].LARVSVLDLK.[K]	1xPhospho [S5(100)]	S414
	High	[K].IKQLELEITELK.[L]		
	High	[R].VSVLDLKK.[I]		
	High	[R].FMLEVMK.[D]	2xOxidation [M2(100); M6(100)]	
	High	[R].VSVLDLK.[K]	1xPhospho [S2(100)]	S414
	High	[K].KFFNCK.[K]	1xCarbamidomethyl [C5]	
	High	[K].KLTESSAVVATSTSKTEGNSAR.[I]		

b

1 MNSPTMKSEQLTPKLSPMSFCLDDQRNAGSFQNLLNSPTKLKLDTGPIGNSLLYPTSLSKLSELSRGGRSKQRRGSDTMRSVSPIRFQFLNNTPKMLKPE 100

101 YLSQTTSNLPLLSALLKNSKKTTSEGQNSNPDPLNIEKNIIKQSIKDKLEQLRSSESVAQVQKKERNPPSFEAKVCAEEPILRKNAEGLLPSYVPVPATP 200

201 LEDPENHGVRKVEDKGLRVVSGGSTQCLSTEVNELPKDLNLDNLPTDNNGFVQYGLKGNNNNNRYSFISSTSTDYEPEWCDGQQHISMQMASMANAEEAN 300

301 SREKSNLDIKIKQLELEITELKLQNEKLVHSMTTNRYIEERFMLEVMKDPSIQAQRSQRDIERKVKQLEKKFFNCKKVLKKLTESSAVVATSTSKTEGNS 400

401 ARIPCPKTRLARVSVLDLKKIEEQPDSSSGTSSEEDHLTNDDTDANTSEDLNVAFEEEPTSAISTTASVQSGESKRGFQLNLPVQVEKKEK* 491

Supplementary Fig. 4 | Phosphorylation sites in Mmr1 detected by MS analysis

a) Mmr1-derived peptides detected by protein MS analysis are listed with detected modifications. Phosphorylation of Mmr1 at S224 and S414 residues was detected with the best site possibility of 99.6% and 100%, respectively. b) Deduced amino acid sequence of Mmr1. S224 and S414 residues are indicated by cyan and red arrowheads, respectively. Motifs that match to the RxS phosphorylation consensus sequence of Ste20 and Cla4 are boxed.

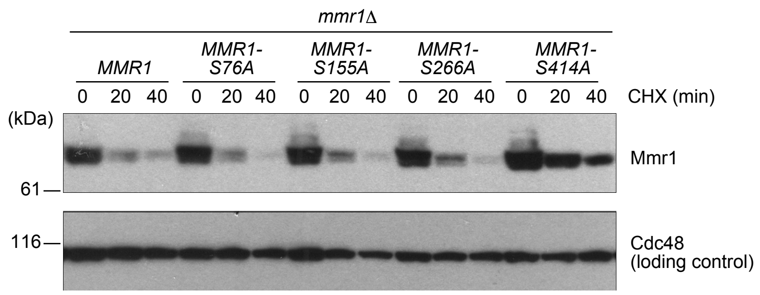

Supplementary Fig. 5 | S414 residue is important for Mmr1 turnover

YTK5777 (*mmr1Δ MMR1*), YTK6316 [*mmr1Δ MMR1(S76A)*], YTK6021 [*mmr1Δ MMR1(S155A)*], YTK5984 [*mmr1Δ MMR1(S266A)*], and YTK5778 [*mmr1Δ MMR1(S414A)*] cells were grown to log phase in YPD medium at 30˚C and subjected to cycloheximide (CHX) chase. Their total lysates were prepared and subjected to immunoblot analysis with anti-Mmr1 or, to demonstrate uniform loading, anti-Cdc48 antibody. Similar results were obtained from two independent experiments.

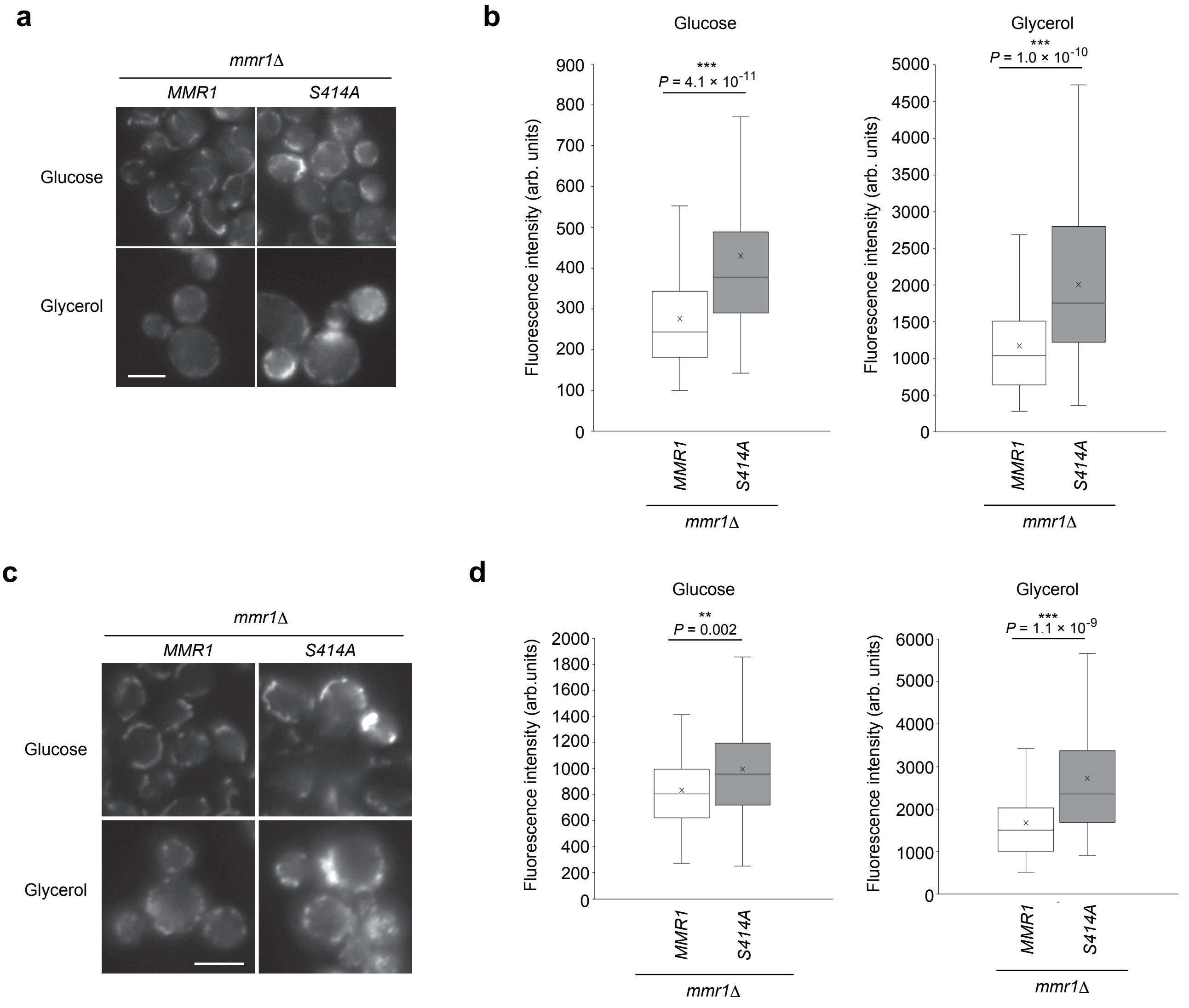

Supplementary Fig. 6 | *MMR1(S414A)* cells have elevated respiratory activity and produce more ROS
a) YTK5675 (*mmr1Δ MMR1*), and YTK5764 [*mmr1Δ MMR1(S414A)*] cells were grown to log phase in synthetic complete medium (either glucose or glycerol was used as the carbon source), loaded with 50 nM TMRM for 1 h, and observed under a fluorescence microscope. Bar, 5 µm. b) Intensity of TMRM signal. Imaging data from (a) were used for the analysis. c) YTK5675 (*mmr1Δ MMR1*), and YTK5764 [*mmr1Δ MMR1(S414A)*] cells were grown to log phase in synthetic complete medium (either glucose or glycerol was used as the carbon source), loaded with 5 µM CellROX Green reagent for 1 h, and observed under a fluorescence microscope. Bar, 5 µm. d) Intensity of CellROX Green reagent signal. Imaging data from (c) were used for the analysis. In (b) and (d), the box covers the region from the 1st quartile to the 3rd quartile. The horizontal line and the cross mark represent the median and mean, respectively. The whiskers at either side of the box extend to 1.5 interquartile ranges from the quartiles. In (b), for cells cultured in glucose-containing medium, 120 and 101 cells for *mmr1Δ MMR1* and *mmr1Δ MMR1(S414A)* cells, respectively, were analyzed, whereas in glycerol-containing medium, 102 and 108 cells for *mmr1Δ MMR1* and *mmr1Δ MMR1(S414A)* cells, respectively, were analyzed. In (d), for cells cultured in glucose-containing medium, 110 cells both for *mmr1Δ MMR1* and *mmr1Δ MMR1(S414A)* cells were analyzed, whereas in glycerol-containing medium, 105 and 102 cells for *mmr1Δ MMR1* and *mmr1Δ MMR1(S414A)* cells, respectively, were analyzed. Significance was tested by Mann-Whitney's *U* test. Similar results were obtained from two independent experiments.

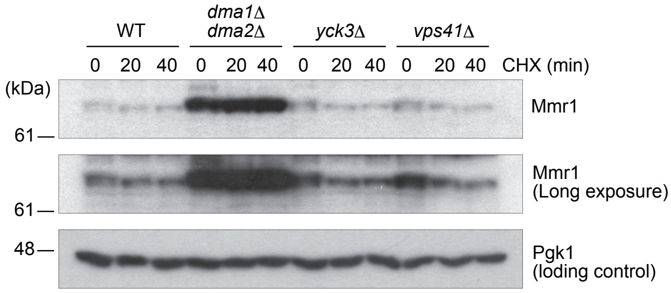

Supplementary Fig. 7 | Yck3 and Vps41 are dispensable for rapid turnover of Mmr1

BY4741 (WT), YOK5261 (*dma1*Δ *dma2*Δ), 6122 (*yck3*Δ), and 4015 (*vps41*Δ) cells grown to log phase in YPD medium at 30˚C were treated with cycloheximide (CHX) and harvested. Their total lysates were prepared and subjected to immunoblot analysis with anti-Mmr1 or, to demonstrate uniform loading, anti-Pgk1 antibody. Similar results were obtained from two independent experiments.

Uncropped gel images

Fig. 1a

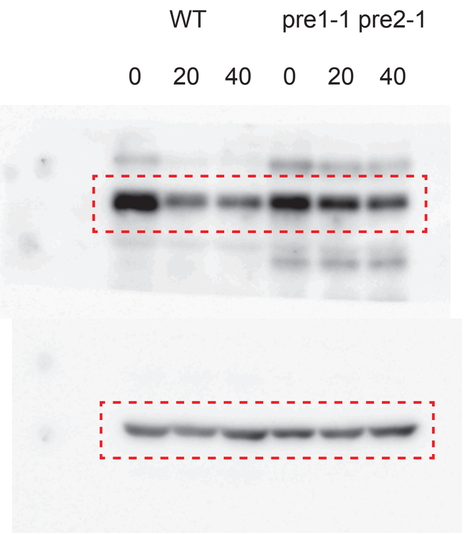

Fig. 1c

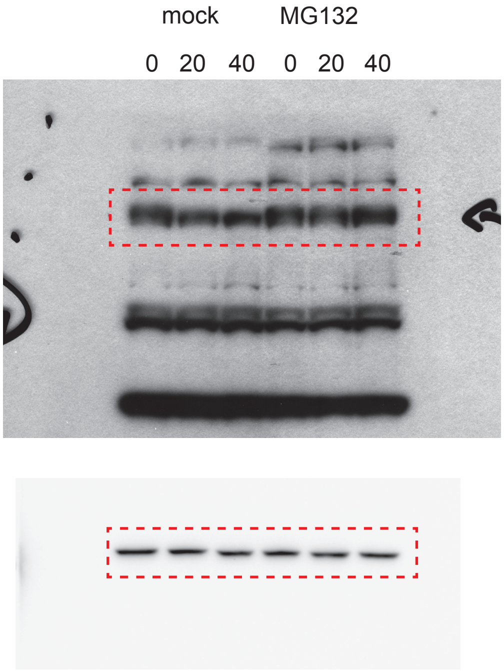

Fig. 2a

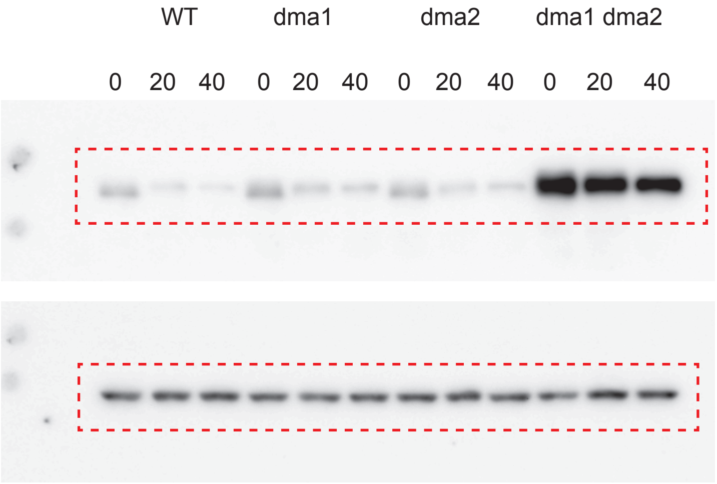

Fig. 2d

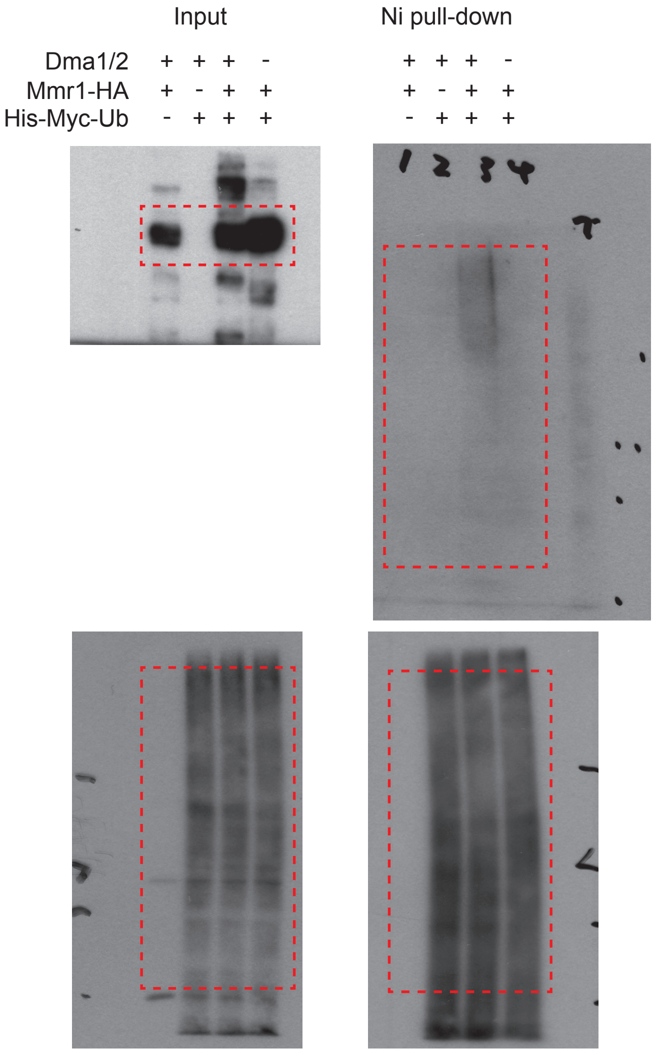

Fig. 2c

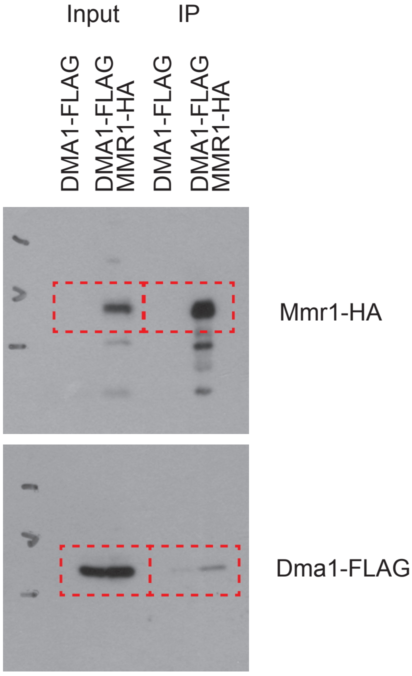

Fig. 5a

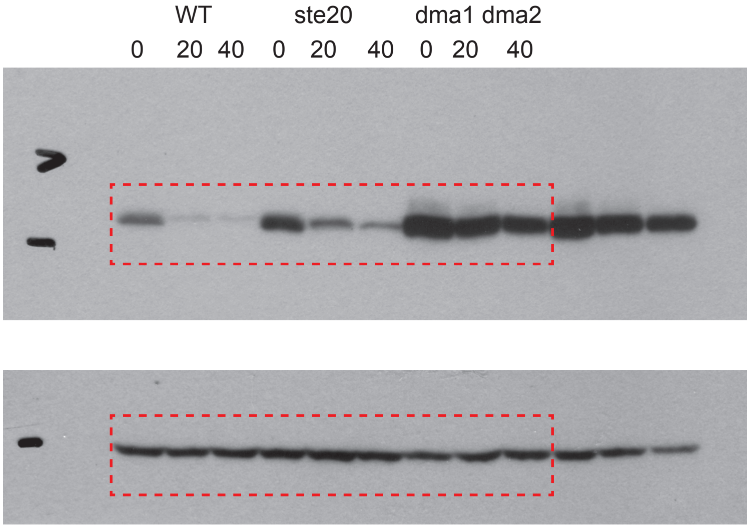

Fig. 5b

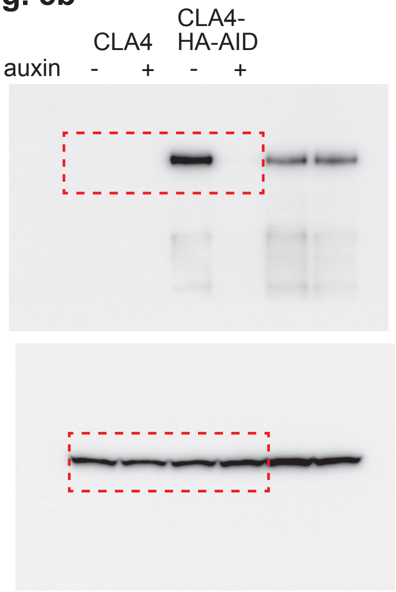

Fig. 5c

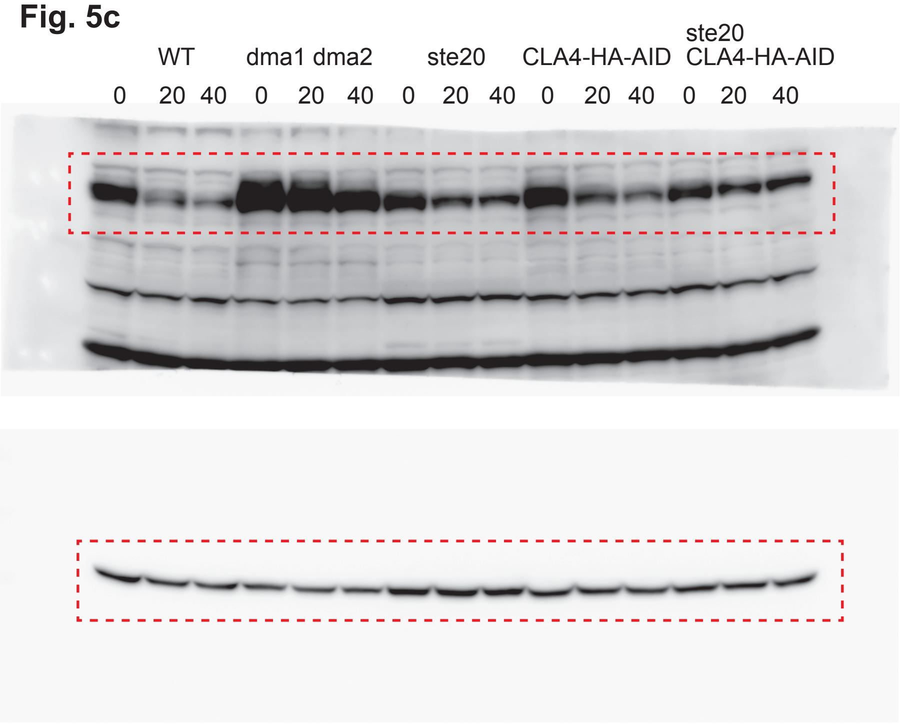

Fig. 5e

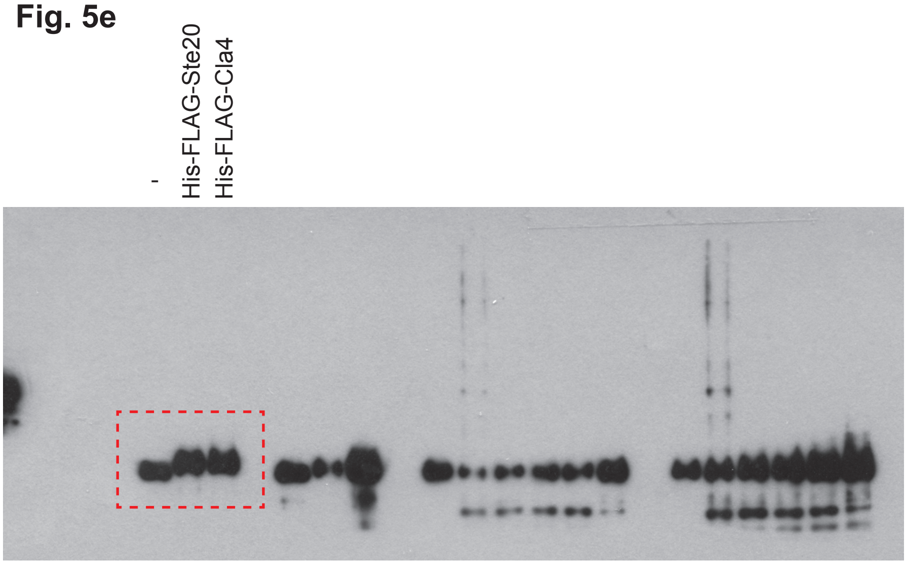

Fig. 5g

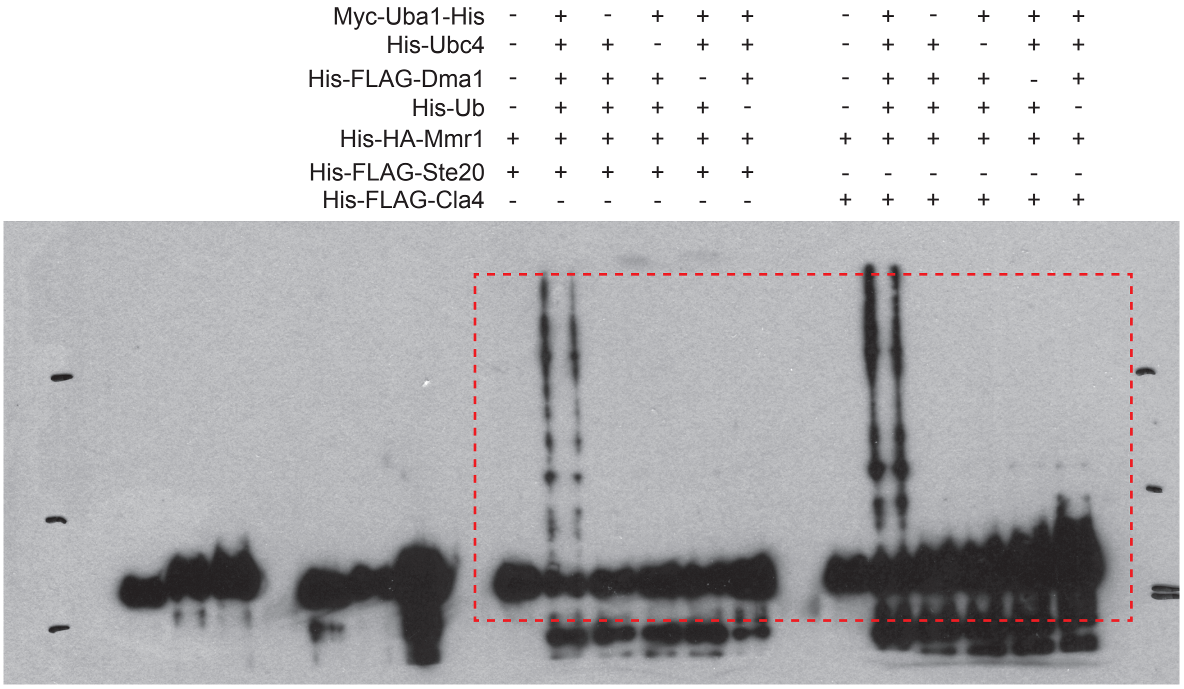
Myc-Uba1-His - + - + + + - + - + + +
His-Ubc4 - + + - + + - + + - + +
His-FLAG-Dma1 - + + + - + - + + + - +
His-Ub - + + + + - - + + + + -
His-HA-Mmr1 + + + + + + + + + + + +
His-FLAG-Ste20 + + + + + + - - - - - -
His-FLAG-Cla4 - - - - - - + + + + + +

Fig. 5h

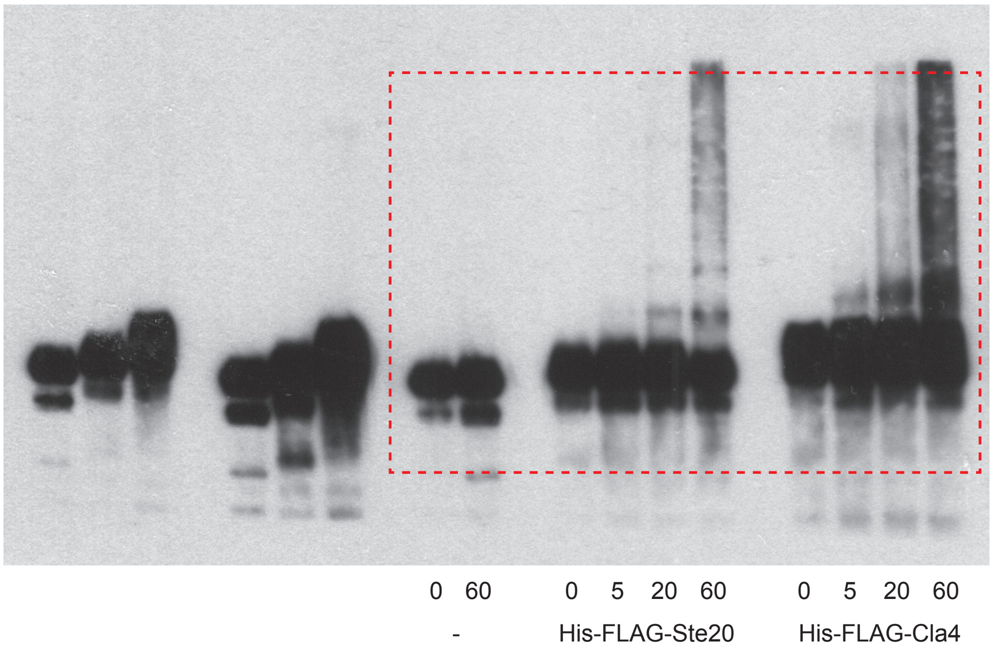
0 60 0 5 20 60 0 5 20 60
- His-FLAG-Ste20 His-FLAG-Cla4

Fig. 6a

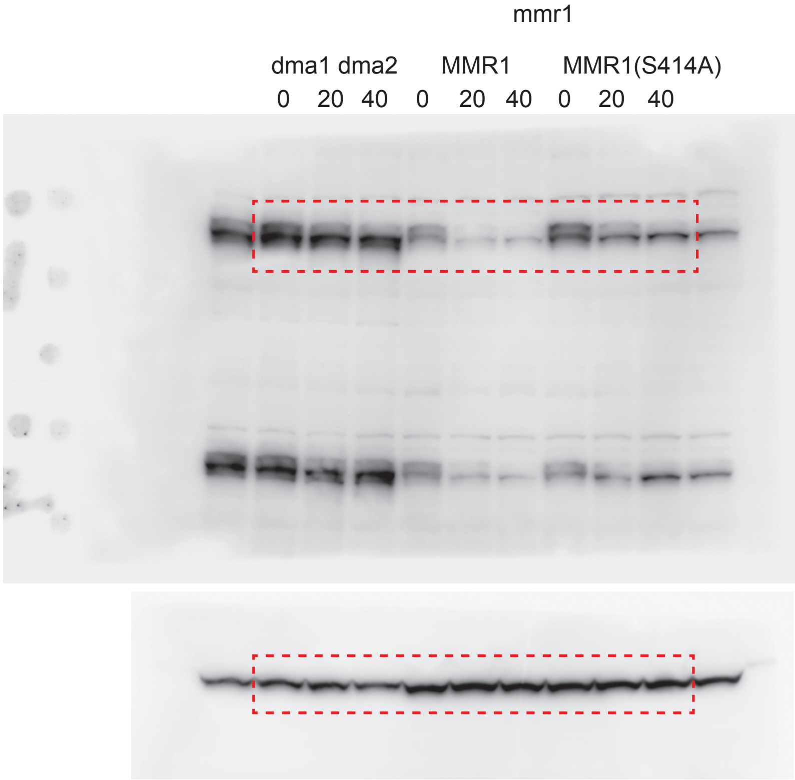
mmr1
dma1 dma2
MMR1
MMR1(S414A)
0 20 40 0 20 40 0 20 40

Fig. 7d

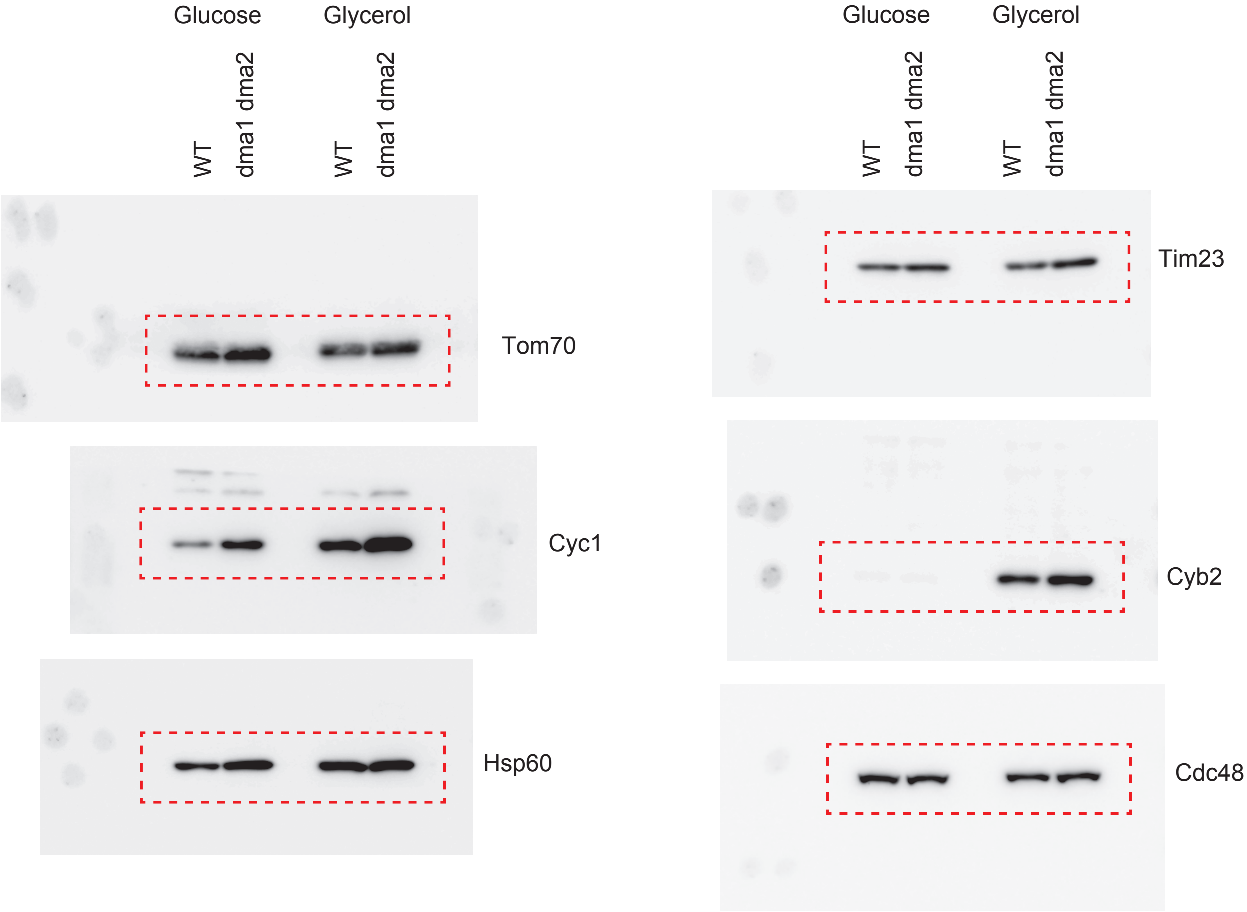
Glucose
Glycerol
WT
dma1 dma2
WT
dma1 dma2
Tom70
Cyc1
Hsp60
Glucose
Glycerol
WT
dma1 dma2
WT
dma1 dma2
Tim23
Cyb2
Cdc48

Supplementary Fig. 1a

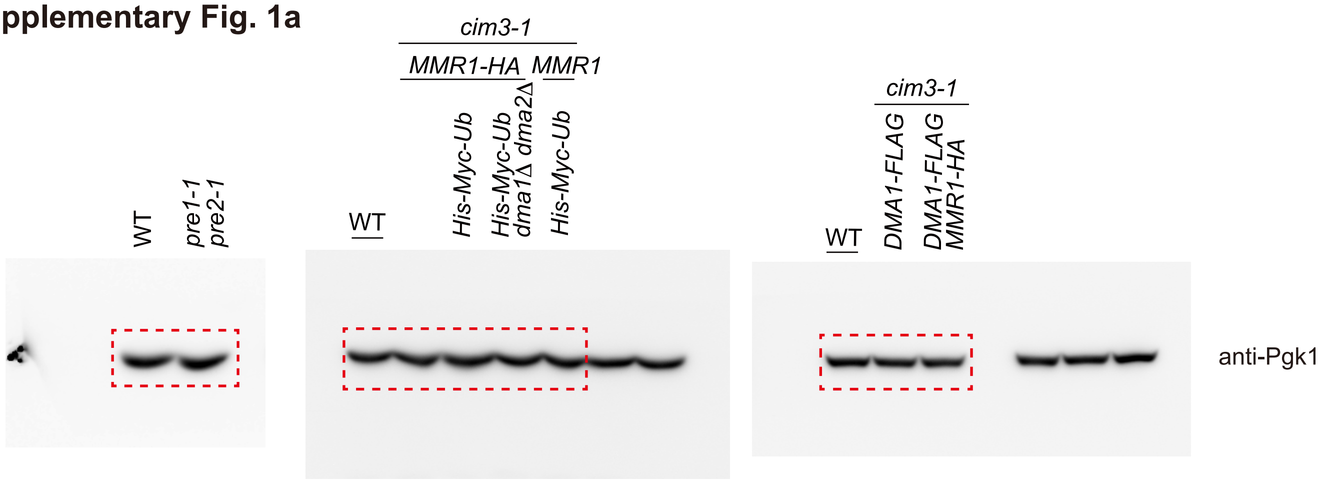

Supplementary Fig. 1b

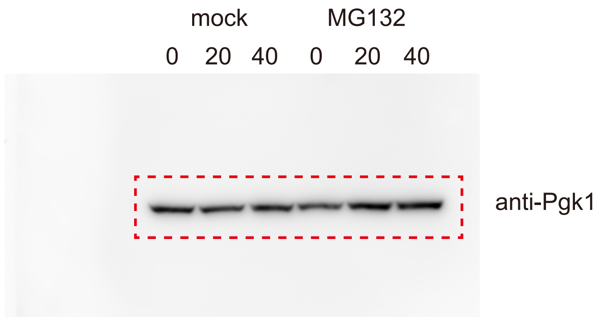

Supplementary Fig. 2a

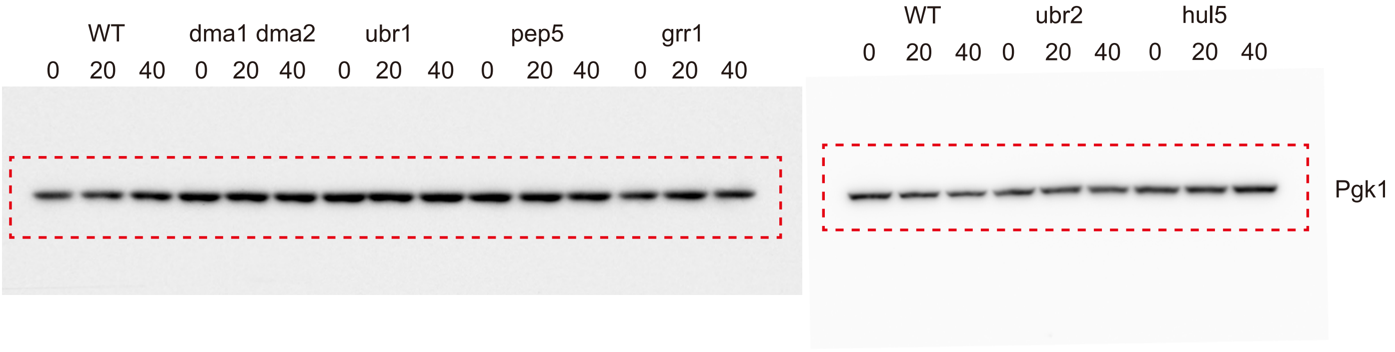

Supplementary Fig. 2b

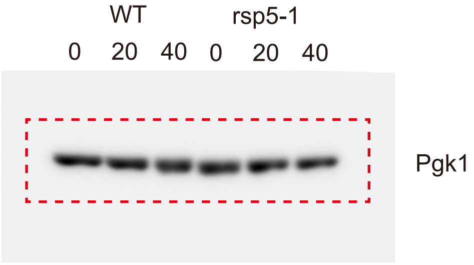

Supplementary Fig. 5

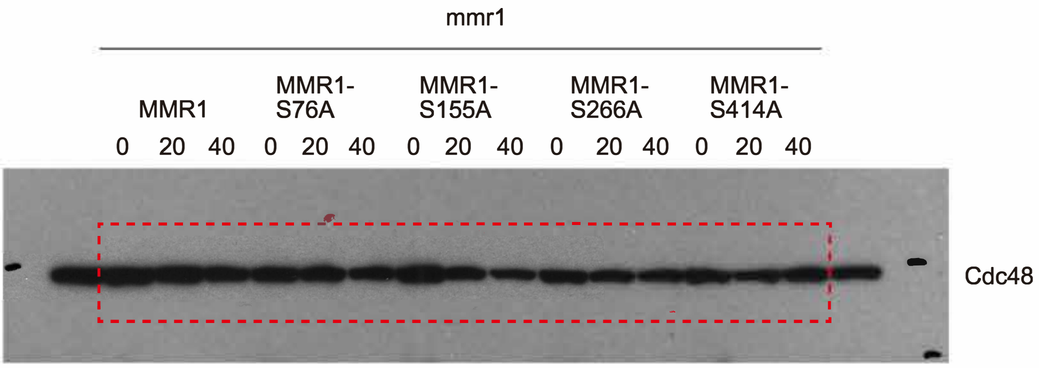

Supplementary Fig. 7

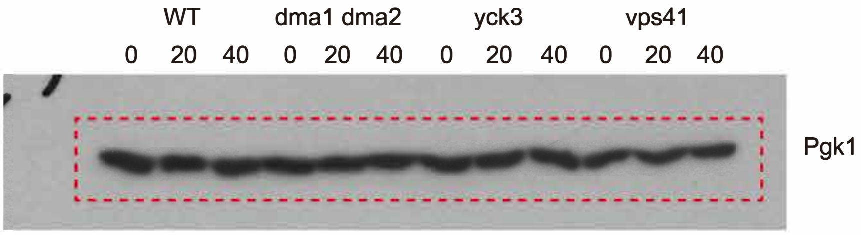

Supplementary References

1. Heinemeyer, W., Gruhler, A., Mohrle, V., Mahe, Y. & Wolf, D.H. PRE2, highly homologous to the human major histocompatibility complex-linked RING10 gene, codes for a yeast proteasome subunit necessary for chrymotryptic activity and degradation of ubiquitinated proteins. *J Biol Chem* **268**, 5115-5120 (1993).
2. Heinemeyer, W., Kleinschmidt, J.A., Saidowsky, J., Escher, C. & Wolf, D.H. Proteinase yscE, the yeast proteasome/multicatalytic-multifunctional proteinase: mutants unravel its function in stress induced proteolysis and uncover its necessity for cell survival. *EMBO J* **10**, 555-562 (1991).
3. Brachmann, C.B. et al. Designer deletion strains derived from *Saccharomyces cerevisiae* S288C: a useful set of strains and plasmids for PCR-mediated gene disruption and other applications. *Yeast* **14**, 115-132 (1998).
4. Sikorski, R.S. & Hieter, P. A system of shuttle vectors and yeast host strains designed for efficient manipulation of DNA in *Saccharomyces cerevisiae*. *Genetics* **122**, 19-27 (1989).
5. Ghislain, M., Udvardy, A. & Mann, C. *S. cerevisiae* 26S protease mutants arrest cell division in G2/metaphase. *Nature* **366**, 358-362 (1993).
6. Winzeler, E.A. et al. Functional characterization of the *S. cerevisiae* genome by gene deletion and parallel analysis. *Science* **285**, 901-906 (1999).
7. Abe, F. & Iida, H. Pressure-induced differential regulation of the two tryptophan permeases Tat1 and Tat2 by ubiquitin ligase Rsp5 and its binding proteins, Bul1 and Bul2. *Mol Cell Biol* **23**, 7566-7584 (2003).
