# Supplementary material for: Proteolysis of adaptor protein Mmr1 during budding is necessary for mitochondrial homeostasis in Saccharomyces cerevisiae
Source: Nat Commun. 2022 Apr 14;13:2005. doi: 10.1038/s41467-022-29704-8 (PMC9010424; doi:10.1038/s41467-022-29704-8)
Supplement: Supplementary file 2 — Description of Additional Supplementary Files [file 41467_2022_29704_MOESM2_ESM.pdf]

File Name: Supplementary Data 1
Description: List of proteins detected in the HA-Mmr1-immunoprecipitated fraction.

File Name: Supplementary Movie 1
Description: YTK5334 (*Mt-GFP*) cells were grown to log phase in synthetic complete medium and serially observed under a fluorescence microscope using glass-base dishes.

File Name: Supplementary Movie 2
Description: YTK5341 (*Mt-GFP dma1*Δ *dma2*Δ) cells were grown to log phase in synthetic complete medium and serially observed under a fluorescence microscope using glass-base dishes.

File Name: Supplementary Movie 3
Description: YTK5334 (*Mt-GFP*) cells were grown to log phase in synthetic complete medium, fixed, and observed under a fluorescence microscope. Optical sections were obtained using a structured illumination microscopy system at an interval of 0.2 µm, deconvoluted and reconstructed into the 3D image.

File Name: Supplementary Movie 4
Description: YTK5341 (*Mt-GFP dma1*Δ *dma2*Δ) cells were grown to log phase in synthetic complete medium, fixed, and observed under a fluorescence microscope. Optical sections were obtained using a structured illumination microscopy system at an interval of 0.2 µm, deconvoluted and reconstructed into the 3D image. Mitochondria are stacked at the bud-tip. Bar, 5 µm.

File Name: Supplementary Movie 5
Description: YTK5341 (*Mt-GFP dma1*Δ *dma2*Δ) cells were grown to log phase in synthetic complete medium, fixed, and observed under a fluorescence microscope. Optical sections were obtained using a structured illumination microscopy system at an interval of 0.2 µm, deconvoluted and reconstructed into the 3D image. Mitochondria are stacked at the bud-neck. Bar, 5 µm.
