# Supplementary material for: Proteolysis of adaptor protein Mmr1 during budding is necessary for mitochondrial homeostasis in Saccharomyces cerevisiae
Source: Nat Commun. 2022 Apr 14;13:2005. doi: 10.1038/s41467-022-29704-8 (PMC9010424; doi:10.1038/s41467-022-29704-8)
Supplement: Supplementary file 9 — Reporting Summary [file 41467_2022_29704_MOESM9_ESM.pdf]

nature portfolio

Corresponding author(s): Keisuke Obara
Takumi Kamura

Last updated by author(s): Mar 29, 2022

Reporting Summary

Nature Portfolio wishes to improve the reproducibility of the work that we publish. This form provides structure for consistency and transparency in reporting. For further information on Nature Portfolio policies, see our Editorial Policies and the Editorial Policy Checklist.

Statistics

For all statistical analyses, confirm that the following items are present in the figure legend, table legend, main text, or Methods section.

n/a	Confirmed	
☐	☒	The exact sample size (*n*) for each experimental group/condition, given as a discrete number and unit of measurement
☐	☒	A statement on whether measurements were taken from distinct samples or whether the same sample was measured repeatedly
☐	☒	The statistical test(s) used AND whether they are one- or two-sided<br>*Only common tests should be described solely by name; describe more complex techniques in the Methods section.*
☒	☐	A description of all covariates tested
☐	☒	A description of any assumptions or corrections, such as tests of normality and adjustment for multiple comparisons
☐	☒	A full description of the statistical parameters including central tendency (e.g. means) or other basic estimates (e.g. regression coefficient) AND variation (e.g. standard deviation) or associated estimates of uncertainty (e.g. confidence intervals)
☐	☒	For null hypothesis testing, the test statistic (e.g. *F*, *t*, *r*) with confidence intervals, effect sizes, degrees of freedom and *P* value noted<br>*Give P values as exact values whenever suitable.*
☒	☐	For Bayesian analysis, information on the choice of priors and Markov chain Monte Carlo settings
☒	☐	For hierarchical and complex designs, identification of the appropriate level for tests and full reporting of outcomes
☒	☐	Estimates of effect sizes (e.g. Cohen's *d*, Pearson's *r*), indicating how they were calculated

Our web collection on statistics for biologists contains articles on many of the points above.

Software and code

Policy information about availability of computer code

Data collection	Immunoblot images were obtained using the ImageQuant LAS4000 ver1.2 (GE healthcare) software. Micrographs were obtained using ZEN 3.3 pro (Zeiss) or AxioVision 4.8.2 (Zeiss).
Data analysis	Proteome Discoverer 2.2.0.388 (ThermoFisher Scientific) and the SEQUEST (ThermoFisher Scientific) softwares were used for the analysis of LC-MS/MS experiments. Photoshop CS3 (Adobe) was used to process immunoblot images and micrographs. Image J (version 1.52 or 1.53) was used to analyze the fluorescence intensity of TMRM and CellROX Green reagents. ImageQuant TL v8.1 (GE healthcare) was used to quantify the signal intensity in immunoblot analysis. R version 4.1.0 was used for statistic analysis.

For manuscripts utilizing custom algorithms or software that are central to the research but not yet described in published literature, software must be made available to editors and reviewers. We strongly encourage code deposition in a community repository (e.g. GitHub). See the Nature Portfolio guidelines for submitting code & software for further information.

Data

Policy information about availability of data

All manuscripts must include a data availability statement. This statement should provide the following information, where applicable:

- Accession codes, unique identifiers, or web links for publicly available datasets
- A description of any restrictions on data availability
- For clinical datasets or third party data, please ensure that the statement adheres to our policy

The LC-MS/MS data generated in this study have been deposited in the jPOST database under accession code PXD032866.
Source data are provided for Figures 1b, 1d, 2b, 5d, 6b, and 7e (immunoblot); Figures 3b, 3c, 5j, and 6d (measurement of mitochondria stack); Figures 7c, 8b, and Supplementary Figure 7b and 7d (measurement of fluorescence intensity); and Figure 8e (measurement of cell death).

Data in this manuscripts are available from corresponding authors on reasonable requests.

Field-specific reporting

Please select the one below that is the best fit for your research. If you are not sure, read the appropriate sections before making your selection.

[x] Life sciences [ ] Behavioural & social sciences [ ] Ecological, evolutionary & environmental sciences

For a reference copy of the document with all sections, see nature.com/documents/nr-reporting-summary-flat.pdf

Life sciences study design

All studies must disclose on these points even when the disclosure is negative.

Sample size	No statistical methods were used to predetermine the sample size. All cell-based experiments were performed with yeast clones. Number of cells analyzed (n > 100) for the fluorescence measurement analysis (Figs. 7C and 8b, and Supplementary Fig 7b and d) was chosen based on our preliminary experiments that have shown robust consistency between assays, n of 100 or more were established as sufficient to reveal differences between clones. For cycloheximide chase and measurement of mitochondria stack, data from three independent experiments were established, by our previous studies and preliminary experiments, as sufficient to show differences between clones.
Data exclusions	No data was excluded.
Replication	Cycloheximide chase assay (Figs 1, 2, 5 and 6) was repeated three times. Cell growth assay on plates (Figs. 7 and 8) were performed at least twice. Quantification of mitochondria stacking (Figs. 3, 5, and 6) was performed after three independent experiments. Test for susceptibility to ethanol (Fig. 8) was performed three times. Western blotting to quantify the levels of mitochondria proteins (Fig. 7) was performed three times. TMRM assay and CellROX Green assay (Figs 7 and 8 and Supplementary Fig. 7) were performed twice. All attempts at replication were successful.
Randomization	This is not relevant to our study. Our study compared lifetime of Mmr1 protein, mitochondria dynamics, and mitochondria function of yeast clones in which different genes were deleted or modified in parallel.
Blinding	This is not relevant to our study. Our study compared lifetime of Mmr1 protein, mitochondria dynamics, and mitochondria function of yeast clones in which different genes were deleted or modified in parallel.

Reporting for specific materials, systems and methods

We require information from authors about some types of materials, experimental systems and methods used in many studies. Here, indicate whether each material, system or method listed is relevant to your study. If you are not sure if a list item applies to your research, read the appropriate section before selecting a response.

Materials & experimental systems

n/a	Involved in the study	
[ ]	[x]	Antibodies
[ ]	[x]	Eukaryotic cell lines
[x]	[ ]	Palaeontology and archaeology
[x]	[ ]	Animals and other organisms
[x]	[ ]	Human research participants
[x]	[ ]	Clinical data
[x]	[ ]	Dual use research of concern

Methods

n/a	Involved in the study	
[x]	[ ]	ChIP-seq
[x]	[ ]	Flow cytometry
[x]	[ ]	MRI-based neuroimaging

Antibodies

Antibodies used

Polyclonal rabbit anti-Mmr1 antibody; Dilution 1:200; Prepared in our laboratory
Monoclonal mouse anti-Pgk1 antibody (22C5D8, ThermoFisher Scientific, UA2696317); Dilution 1:3,000
Monoclonal mouse anti-HA antibody (12CA5, Sigma, 11666606001); Dilution 1:5,000
Monoclonal mouse anti-HA antibody (TANA2, Medical and Biological Laboratories, M180-3); Dilution 1:2,000
Monoclonal mouse anti-FLAG antibody conjugated with peroxidase (M2, Sigma, A8592); Dilution 1:10,000
Polyclonal rabbit anti-Myc antibody; Dilution 1:5,000; Prepared in our laboratory
Polyclonal rabbit anti-Tom70; Dilution 1:4,000; Kind gift from Dr. Y. Tamura (Yamagata University); Prepared by them.
Polyclonal rabbit anti-Tim23; Dilution 1:2,000; Kind gift from Dr. Y. Tamura (Yamagata University); Prepared by them.
Polyclonal rabbit anti-Cyc1; Dilution 1:2,000; Kind gift from Dr. Y. Tamura (Yamagata University); Prepared by them.
Polyclonal rabbit anti-Cyb2; Dilution 1:2,000; Kind gift from Dr. Y. Tamura (Yamagata University); Prepared by them.
Polyclonal rabbit anti-Hsp60; Dilution 1:4,000; Kind gift from Dr. Y. Tamura (Yamagata University); Prepared by them.
Polyclonal rabbit anti-Cdc48; Dilution 1:10,000; Prepared in our laboratory
HRP-conjugated anti-mouse IgG (Sigma, A4416); Dilution 1:7,500

	HRP-conjugated anti-rabbit IgG (Sigma, A6154); Dilution 1:7,500
Validation	In immunoblot analysis using yeast lysates, anti-Mmr1 antibody recognizes endogenous Mmr1 with some non-specific bands. We confirmed that Mmr1 bands shown in our manuscript are bona fide Mmr1 signals by comparing with band patterns of samples from MMR1-deleted yeast cells. For anti-HA antibodies, 12CA5 and TANA2, refer to https://www.sigmaaldrich.com/JP/en/product/roche/roaha and https://ruo.mbl.co.jp/bio/e/dtl/A/?pcd=M180-3, respectively. For anti-FLAG antibody, refer to https://www.sigmaaldrich.com/JP/en/product/sigma/a8592. In immunoblot analysis of yeast lysates, anti-Myc antibody recognizes Myc-tagged proteins but not untagged proteins. Bands in Fig. 2 detected by anti-HA, anti-FLAG, and anti-Myc antibodies were confirmed to be specific signals by comparing with control samples in the same panel. For anti-Pgk1 antibody, refer to https://www.thermofisher.com/antibody/product/PGK1-Antibody-clone-22C5D8-Monoclonal/459250. For anti-Tom70, anti-Tim23, anti-Cyc1, and anti-Cyb2 antibodies, refer to https://www.sciencedirect.com/science/article/pii/S2211124718320151?via%3Dihub. For anti-Hsp60 antibody, refer to https://rupress.org/jcb/article/185/6/1029/35183/Ups1p-and-Ups2p-antagonistically-regulate.<br>In immunoblot analysis of yeast lysates, anti-Cdc48 antibody recognizes endogenous Cdc48 the band of which is upshifted when Cdc48 was chromosomally tagged with GFP. For anti-Cdc48 antibody, also refer to https://www.sciencedirect.com/science/article/pii/S1097276515002695.

Eukaryotic cell lines

Policy information about cell lines

Cell line source(s)	Sf21 cells were used for protein expression (ThermoFisher Scientific, B82101).
Authentication	Sf21 cells were not formally authenticated. However, Sf21 cells were susceptible to baculovirus infection and expressed recombinant proteins encoded by these baculovirus, which is consistent with their authenticity.
Mycoplasma contamination	Sf21 cell line was not tested for mycoplasma contamination.
Commonly misidentified lines (See ICLAC register)	Nil
